# Integrative Phylogenetic and Morphological Analyses Reveal Two New Species of Porcellanid Crabs and Resurrect *Porcellanella picta* Stimpson, 1858 (Decapoda: Porcellanidae)

**DOI:** 10.1002/ece3.72131

**Published:** 2025-09-29

**Authors:** Hai Xin Loke, Bonnie Yuen Wai Heung, Yi‐Xuan Li, Yi‐Tao Lin, Andrew M. Hosie, Zhi Wang, Marissa McNamara, Jian‐Wen Qiu

**Affiliations:** ^1^ Department of Biology Hong Kong Baptist University Hong Kong P. R. China; ^2^ Department of Aquatic Zoology Western Australian Museum Welshpool Western Australia Australia; ^3^ State Key Laboratory of Marine Environmental Science, College of Ocean and Earth Sciences Xiamen University Xiamen P. R. China; ^4^ Collections and Research Centre Queensland Museum Brisbane Queensland Australia

**Keywords:** Anomura, biogeography, cryptic species, morphology, phylogeny

## Abstract

Members of the genus *Porcellanella* White, 1851 (Porcellanidae) are common commensals of sea pens in tropical and subtropical coastal waters. Despite having only three described species ( *P. triloba* White, 1851 from Cape Capricorn, Australia; *P. picta* Stimpson, 1858 from Hong Kong; and *P. haigae* Sankarankutty, 1963 from the Gulf of Mannar, Indian Ocean), the taxonomy of this genus has been widely debated. *Porcellanella picta* was previously considered a junior synonym of *P. triloba* , but this synonymization has been the subject of disagreement. In this study, we conducted integrative phylogenetic and morphological analyses of *Porcellanella* specimens tentatively identified as *P. triloba* , collected from Hong Kong, Taiwan, mainland China, Thailand, Singapore and Australia. Our Maximum Likelihood and Bayesian Inference phylogenetic analyses of mitochondrial *COI* and *16S rRNA* sequences revealed four distinct lineages: one corresponding to *P. picta* from Asia, one to *P. triloba* and two representing new species (*P. brevidentata* n. sp. and *P. longiloba* n. sp.) from Australia. Morphologically, *Porcellanella* species can be distinguished by the colour markings on the chelipeds and carapace, the shape of the trilobate rostrum, the presence or absence of a meral lobe on the cheliped, the relative size of the unguicles on the ambulatory leg dactylus, and the presence or absence of a spinule on the lateral margin of the pterygostomian flap. We provide a morphological key to the species of *Porcellanella*. Our study demonstrates the value of an integrative approach in distinguishing cryptic invertebrate species that are considered to exhibit wide geographic distribution patterns.

## Graphical abstract

This study uses integrative phylogenetic and morphological analyses to clarify the taxonomy of Porcellanella crabs, revealing four distinct species. The authors resurrect Porcellanella picta as a valid species and describe two new species from Australia (*P. brevidentata* n. sp. and *P. longiloba* n. sp.). Their findings highlight the importance of combined genetic and morphological approaches in uncovering cryptic diversity among widely distributed invertebrates.

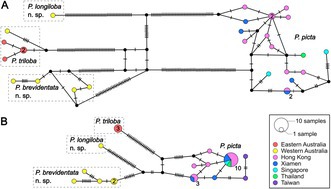

## 1 Introduction

Many marine invertebrate species occupy extensive geographic ranges (Scheltema 1971). These broad distributions were thought to result from the ocean's high uniformity and the absence of significant physical barriers to dispersal (Madsen 1961), along with prolonged larval dispersal phases of up to 10 months in certain species (Scheltema 1971). However, subsequent research on these widely distributed organisms has produced varied findings. While many studies have identified cryptic species—organisms that look alike but are genetically distinct (Ragionieri et al. 2009; Nygren and Pleijel 2011; Capa et al. 2013; Fang et al. 2023), a small number of studies have confirmed the wide ranges of these species (Georgieva et al. 2015; McCowin et al. 2019). Nonetheless, for many invertebrates, a thorough evaluation of their species identity remains lacking, complicating our understanding of biodiversity and biogeographic patterns (Fišer et al. 2018; Hutchings and Kupriyanova 2018).

The porcelain crab genus *Porcellanella* White, 1851 (Crustacea: Porcellanidae) is a notable example of a marine taxon whose species diversity and distribution patterns are poorly studied. Members of this genus are common commensals of sea pens in tropical and subtropical coastal waters (Osawa and McLaughlin 2010). Although small, with a carapace length of less than 15 mm, these crabs have been well‐noted by SCUBA divers due to their prominent colour patches on the carapace and cheliped claws, with 204 records from the Indo‐Pacific region on the iNaturalist website (iNaturalist Community 2024). The taxonomy of this genus is complex, although only three species have been described: *P. triloba* White, (1851) from northeastern Australia; *P. picta* Stimpson, (1858) from Hong Kong; and *P. haigae* Sankarankutty, (1963) from the Gulf of Mannar, Indian Ocean. Miyake (1943) and Johnson (1964) considered *P. picta* and *P. triloba* distinct species, but Henderson (1893); Sankarankutty (1961) and Haig (1992) treated *P. picta* as a junior synonym of *P. triloba* . Outside their type localities, *P. triloba* has been recorded from as far as the Falkland Islands and Zanzibar (Barnard 1950); *P. picta* has been recorded from the Indian Ocean (Sivasubramanian et al. 2014). However, Sankarankutty (1961) previously identified *Porcellanella* specimens from the Indian Ocean to be *P. triloba* , while Johnson (1964) considered them a subspecies of *P. picta* .

Despite the taxonomic confusion, no phylogeny has been conducted on the genus *Porcellanella*. Before this study, only one molecular marker sequence of *Porcellanella* was available in GenBank: accession number EU834069—a partial *16S rRNA* gene of a specimen sampled from Taiwan and identified as *P. triloba* (assessed on January 3, 2024). This sequence was generated during a phylogenetic study of anomurans (Ahyong et al. 2009).

This taxonomic issue became evident during a benthic ecology study (Ip et al. 2024) conducted in the water body between Lantau Island and Hong Kong Island in 2022 (see Figure S1 for sampling stations). Our bottom trawling and SCUBA diving surveys revealed that *Porcellanella* crabs are common commensals of sea pens, typically residing among the polyp leaves of *Pteroeides sparmannii* Kölliker, 1869 and occasionally associated with *Virgularia* spp., at depths ranging from 10 to 30 m. However, local literature contains conflicting information, with some researchers identifying specimens of *Porcellanella* as *P. picta* (Morton and Morton 1983; Morton, 1988), and others as *P. triloba* (Haig, 1992). To resolve this taxonomic confusion, we initiated integrative morphological and molecular phylogenetic analyses based on samples collected from the type localities of *P. picta* (Hong Kong) and *P. triloba* (Australia), as well as from non‐type localities of Taiwan, mainland China, Thailand, Singapore and Australia. Considering the wide geographical range covered by the involved specimens, we hypothesised that the species located in Hong Kong and Australia are distinct taxonomic entities.

## 2 Materials and Methods

### 2.1 Samples

**FIGURE 1:**
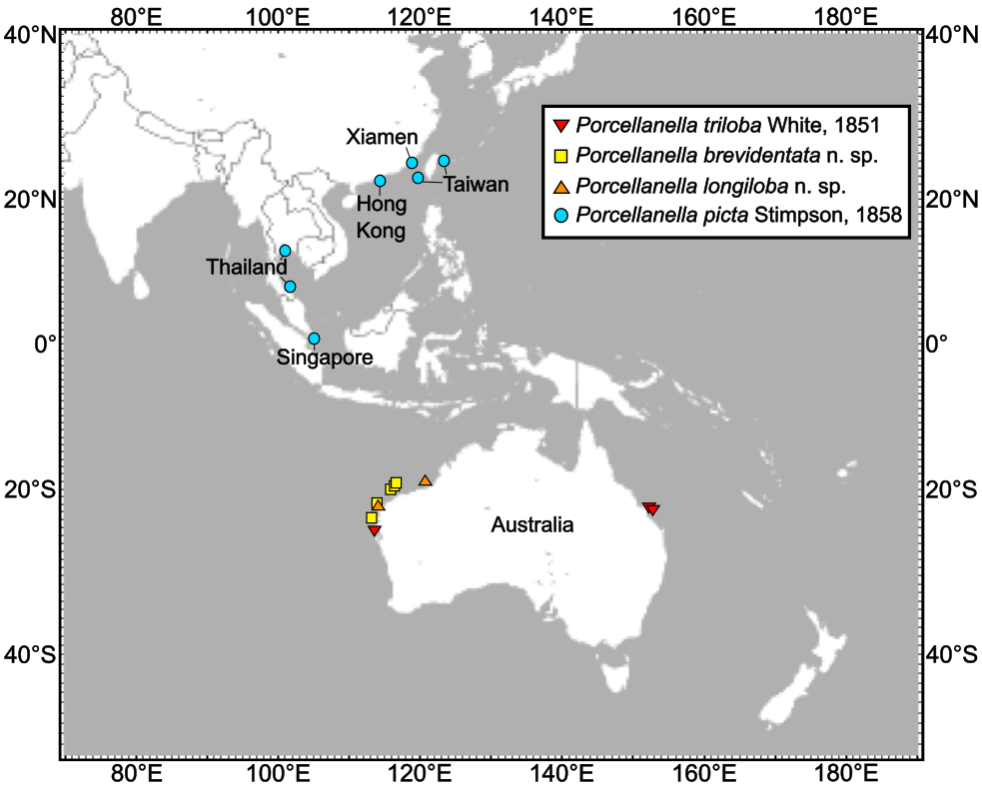
Geographic distribution of *Porcellanella* specimens across Australia and Asia examined during this study. Red inverted triangle: *P. triloba* White, (1851); yellow square: *P. brevidentata* n. sp.; orange triangle: *P. longiloba* n. sp.; cyan circle: *P. picta* Stimpson, (1858). The map was downloaded from OpenStreetMap (https://www.openstreetmap.org/copyright).

**FIGURE 2:**
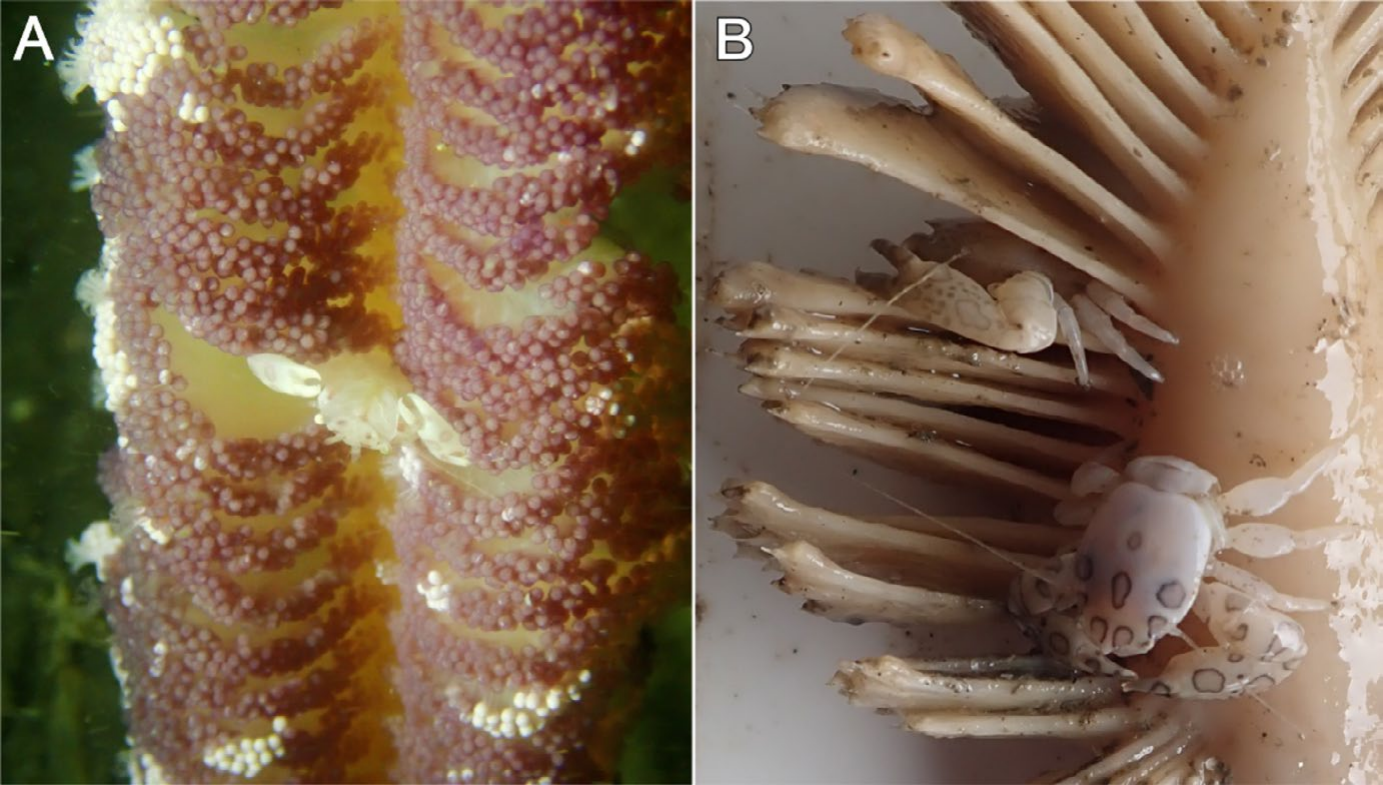
Images of porcelain crab (*Porcellanella picta*) found residing among the polyp leaves of sea pens: (A) *Virgularia halisceptrum*. Location: Siu A Chau, south of Lantau Island, Hong Kong. Date: 2 September 2022; and (B) *Pteroeides sparmannii*. Location: Lung Kwu Chau, north of Lantau Island, Hong Kong. Date: 5 March 2024.

A total of 47 specimens of *Porcellanella* were examined (Table 1). They were collected from Hong Kong, Taiwan, Xiamen, Thailand, Singapore, Australia and preserved in 90% ethanol (Figure 1). Specimens of *P. picta* collected from Hong Kong (Figure 2) and Xiamen have been deposited in the Tropical Marine Biodiversity Collections of South China Sea, Chinese Academy of Sciences, Guangzhou, China (SCSMBC); Marine Biological Museum, Chinese Academy of Sciences, Qingdao, China (MBM); and State Key Laboratory of Marine Environmental Science, Xiamen University, Xiamen, China (XMU‐Art). Specimens collected from other regions were loaned from the Lee Kong Chian Natural History Museum, National University of Singapore, Singapore (ZRC); Western Australian Museum, Welshpool, Australia (WAMC); and Queensland Museum, Queensland, Australia (QMC).

**TABLE 1:** Specimens used in this study. Locality abbreviations: EA, eastern Australia; HK, Hong Kong; SG, Singapore; TL, Thailand; TW, Taiwan; WA, western Australia; XM, Xiamen.

Species	Isolate	Voucher	Collection date	Locality	Latitude	Longitude	DNA extract
*Porcellanella triloba*	EA01	QMC527357.3	10‐Nov‐2005	Great Barrier Reef, EA	22°42′54.0″S	150°57′54.0″ E	Y
	EA02	QMC527357.2	10‐Nov‐2005	Great Barrier Reef, EA	22°42′54.0″S	150°57′54.0″ E	Y
	EA03	QMC527357.1	10‐Nov‐2005	Great Barrier Reef, EA	22°42′54.0″S	150°57′54.0″ E	Y
	EA04	QMC518947.1	19‐Sep‐2004	Great Barrier Reef, EA	22°44′42.0″S	150°51′18.0″ E	Y
	EA05	QMC518947.2	19‐Sep‐2004	Great Barrier Reef, EA	22°44′42.0″S	150°51′18.0″ E	N
	WA02	WAMC74400	25‐Feb‐2019	Shark Bay, WA	24°56′01.0″S	113°23′34.0″ E	Y
*Porcellanella picta*	HK01	SCSMBC240190	10‐Jun‐2022	Hei Ling Chau, HK	22°15′31.7″N	114°02′26.4″ E	Y
	HK02	SCSMBC240189	25‐Apr‐2022	Lamma East, HK	22°11′21.4″N	114°10′05.2″ E	Y
	HK03	SCSMBC240197	13‐Apr‐2022	Lamma Off, HK	22°10′06.1″N	114°10′55.9″ E	Y
	HK04	SCSMBC240187	13‐Apr‐2022	Lamma Off, HK	22°10′06.1″N	114°10′55.9″ E	Y
	HK05	SCSMBC240186	13‐Apr‐2022	Lamma Off, HK	22°10′06.1″N	114°10′55.9″ E	Y
	HK06	SCSMBC240192	22‐Mar‐2023	Kau Yi Chau South, HK	22°16′18.8″N	114°04′44.9″ E	Y
	HK07	SCSMBC240191	10‐Jun‐2022	Hei Ling Chau, HK	22°15′31.7″N	114°02′26.4″ E	Y
	HK08	SCSMBC240188	13‐Apr‐2022	Lamma Off, HK	22°10′06.1″N	114°10′55.9″ E	Y
	HK09	SCSMBC240196	22‐Mar‐2023	Shek Kwu Chau, HK	22°11′41.2″N	114°00′13.8″ E	Y
	HK10	SCSMBC240185	13‐Apr‐2022	Lamma Off, HK	22°10′06.1″N	114°10′55.9″ E	Y
	HK11	SCSMBC240195	22‐Mar‐2023	Kau Yi Chau South, HK	22°16′18.8″N	114°04′44.9″ E	Y
	HK12	SCSMBC240194	22‐Mar‐2023	Lamma Off, HK	22°10′06.1″N	114°10′55.9″ E	Y
	HK13	SCSMBC240193	22‐Mar‐2023	Lamma Off, HK	22°10′06.1″N	114°10′55.9″ E	N
	HK14	SCSMBC240198	12‐Jul‐2022	Shek Kwu Chau, HK	22°11′41.2″N	114°00′13.8″ E	N
	HK15	SCSMBC240199	12‐Jul‐2022	Shek Kwu Chau, HK	22°11′41.2″N	114°00′13.8″ E	N
	HK16	SCSMBC240200	12‐Jul‐2022	Shek Kwu Chau, HK	22°11′41.2″N	114°00′13.8″ E	N
	SG01	ZRC1998.0115.1	12‐Mar‐1998	Changi Beach, SG	N/A	N/A	Y
	SG02	ZRC2011.0699.1	Aug‐2011	Pulau Tekong, SG	N/A	N/A	Y
	SG03	ZRC2011.0699.2	Aug‐2011	Pulau Tekong, SG	N/A	N/A	Y
	SG04	ZRC1998.0113	12‐Mar‐1998	Bedok Jetty, SG	N/A	N/A	N
	SG05	ZRC1998.0115.2	12‐Mar‐1998	Changi Beach, SG	N/A	N/A	N
	TL01	ZRC2003.0155	6‐Jun‐2003	Pattani fish port, TL	N/A	N/A	Y
	TL02	ZRC2000.0907.1	20‐Feb‐2000	Angsila fish port, TL	N/A	N/A	N
	TW01	ZRC1998.0456.1	25‐May‐1998	Fish port at Tashi, TW	N/A	N/A	Y
	XM01	MBM288154	2‐Jul‐2022	Shuangyu Island East, XM	24°22′56.1″N	118°06′01.0″ E	Y
	XM02	XMU‐Art‐2025‐001	2‐Jul‐2022	Shuangyu Island East, XM	24°22′56.1″N	118°06′01.0″ E	Y
	XM03	XMU‐Art‐2025‐002	2‐Jul‐2022	Shuangyu Island East, XM	24°22′56.1″N	118°06′01.0″ E	Y
	XM04	MBM288153	2‐Jul‐2022	Shuangyu Island East, XM	24°22′56.1″N	118°06′01.0″ E	Y
*Porcellanella brevidentata* n. sp.	WA03	WAMC86754	13‐Dec‐2022	Gascoyne Marine Park, WA	23°54′25.9″S	113°05′51.6″ E	Y
	WA04	WAMC86755	13‐Dec‐2022	Gascoyne Marine Park, WA	23°54′25.9″S	113°05′51.6″ E	Y
	WA05	WAMC86756	13‐Dec‐2022	Gascoyne Marine Park, WA	23°54′25.9″S	113°05′51.6″ E	Y
	WA06	WAMC73615	5‐Nov‐2017	North West Shelf, WA	20°11′56.4″S	115°47′13.2″ E	Y
	WA07	WAMC86753	5‐Nov‐2017	North West Shelf, WA	20°11′56.4″S	115°47′13.2″ E	Y
	WA08	WAMC73616	2‐Nov‐2017	North West Shelf, WA	20°10′49.1″S	15°57′00.7″ E	Y
	WA09	WAMC73614	1‐Nov‐2017	Trimouille Island, WA	20°11′42.7″S	115°57′26.3″ E	Y
	WA10	WAMC44990.1	31‐Jan‐2008	Ningaloo Marine Park, WA	21°48′03.7″S	114°00′14.1″E	N
	WA11	WAMC44990.2	31‐Jan‐2008	Ningaloo Marine Park, WA	21°48′03.7″S	114°00′14.1″ E	N
	WA12	WAMC79506.1	13‐Dec‐2022	Gascoyne Marine Park, WA	23°54′25.9″S	113°05′51.6″ E	N
	WA13	WAMC79506.2	13‐Dec‐2022	Gascoyne Marine Park, WA	23°54′25.9″S	113°05′51.6″ E	N
*Porcellanella longiloba* n. sp.	WA01	WAMC74721	16‐May‐2019	Off Eighty Mile Beach, WA	18°49′16.8″S	120°19′26.3″ E	Y
	WA14	WAMC40916	28‐Apr‐2006	Ningaloo Marine Park, WA	22°10′41.0″S	113°47′33.0″ E	N

### 2.2 Molecular Phylogenetics

A total of 34 specimens were used for molecular analysis (Table 2). For each specimen, one pereopod (ambulatory leg) or pleopod (abdominal appendage) was subsampled for genomic DNA extraction using the DNeasy Blood and Tissue Kit (QIAGEN). The primer pairs LCO1490 and HCO2198 (Folmer et al. 1994) were used to amplify a fragment of the mitochondrial cytochrome oxidase I (*COI*) gene, while 16SAR‐L and 16SBR‐H were used to amplify a fragment of the mitochondrial *16S rRNA* gene (Palumbi et al. 1991). The PCR protocols followed those described by Zhang et al. (2018).

**TABLE 2:** GenBank accession numbers of the gene fragments used in the phylogenetic analyses, genetic distance calculations and haplotype networking.

Species	COI	16 S	Isolate of this study/source of other studies
*Porcellanella triloba*	PQ856283	N/A	QMC527357.3
*Porcellanella triloba*	PQ856282	PQ865373	QMC527357.2
*Porcellanella triloba*	PQ856281	PQ865372	QMC527357.1
*Porcellanella triloba*	PQ856284	PQ865374	QMC518947.1
*Porcellanella triloba*	PQ856287	N/A	WAMC74400
*Porcellanella picta*	PQ856305	PQ865395	SCSMBC240190
*Porcellanella picta*	PQ856307	PQ865397	SCSMBC240189
*Porcellanella picta*	PQ856303	PQ865393	SCSMBC240197
*Porcellanella picta*	PQ856304	PQ865394	SCSMBC240187
*Porcellanella picta*	PQ856302	PQ865392	SCSMBC240186
*Porcellanella picta*	PQ856300	PQ865389	SCSMBC240192
*Porcellanella picta*	PQ856308	PQ865398	SCSMBC240191
*Porcellanella picta*	PQ856306	PQ865396	SCSMBC240188
*Porcellanella picta*	PQ856298	PQ865390	SCSMBC240196
*Porcellanella picta*	PQ856301	PQ865391	SCSMBC240185
*Porcellanella picta*	PQ856299	N/A	SCSMBC240195
*Porcellanella picta*	N/A	PQ865388	SCSMBC240194
*Porcellanella picta*	PQ856297	N/A	ZRC1998.0115.1
*Porcellanella picta*	PQ856296	N/A	ZRC2011.0699.1
*Porcellanella picta*	N/A	PQ865387	ZRC2011.0699.2
*Porcellanella picta*	N/A	PQ865385	ZRC1998.0456.1
*Porcellanella picta*	PQ856295	PQ865386	ZRC2003.0155
*Porcellanella picta*	PQ856291	PQ865381	MBM288154
*Porcellanella picta*	PQ856294	PQ865384	XMU‐Art‐2025‐001
*Porcellanella picta*	PQ856293	PQ865383	XMU‐Art‐2025‐002
*Porcellanella picta*	PQ856292	PQ865382	MBM288153
*Porcellanella longiloba* n. sp.	PQ856286	PQ865378	WAMC74721
*Porcellanella brevidentata* n. sp.	N/A	PQ865377	WAMC86754
*Porcellanella brevidentata* n. sp.	PQ856285	PQ865376	WAMC86755
*Porcellanella brevidentata* n. sp.	PQ856290	PQ865375	WAMC86756
*Porcellanella brevidentata* n. sp.	PQ856289	N/A	WAMC73615
*Porcellanella brevidentata* n. sp.	PQ856288	N/A	WAMC86753
*Porcellanella brevidentata* n. sp.	N/A	PQ865379	WAMC73616
*Porcellanella brevidentata* n. sp.	N/A	PQ865380	WAMC73614
*Porcellanella picta* ^a^	N/A	EU834069	Ahyong et al. (2009)
*Pachycheles monilifer*	ON521187	N/A	Hiller and Werding (2022)
*Pachycheles monilifer*	N/A	DQ865330	Rodríguez et al. (2006)
*Pachycheles pubescens*	MW349544	MW363103	Hultgren et al. (2021)
*Petrolisthes cinctipes*	MW349542	N/A	Hultgren et al. (2021)
*Petrolisthes cinctipes*	N/A	AF260593	Stillman and Reeb (2001)
*Porcellana platycheles*	ON521184	N/A	Hiller and Werding (2022)
*Porcellana platycheles*	N/A	HQ380269	Schnabel et al. (2011)

^a^ The specimen of sequence EU834069 was initially labelled as *Porcellanella triloba* on the NCBI database.

DNA sequences of *Porcellanella* species and outgroups (other genera of the family Porcellanidae) were obtained from GenBank (https://www.ncbi.nlm.nih.gov/) for Maximum Likelihood and Bayesian Inference phylogenetic analyses alongside the new sequences generated in this study (Table 2). The *COI* and *16S rRNA* sequences were analysed using PhyloSuite v1.2.3. software (Zhang et al. 2020), which includes several plugins for various tasks. Sequence alignment was performed with the MAFFT v7.505 plugin (Katoh and Standley 2013) using default settings, followed by alignment cleaning with the trimAI v1.2 plugin (Gutierrez et al. 2009) configured to use eight threads. Model selection was executed through the Concatenation (a mandatory step) and ModelFinder v1.5.4 plugins (Kalyaanamoorthy et al. 2017) with default parameters. Phylogenetic tree reconstruction was conducted using the IQ‐TREE v2.2.0 (Nguyen et al. 2015) and MrBayes v3.2.7 (Ronquist et al. 2012) plugins. For IQ‐TREE, settings included 10,000 bootstrap replicates, the SH‐aLRT branch test enabled, and ran under the GTR + I + G model for both *COI* and *16S rRNA*. MrBayes was configured with 1,000,000 generations, a sampling frequency of 100, 2 runs, 4 chains, a burn‐in fraction of 0.25, and ran under the GTR + I + G + F model for *COI* and the HKY + G + F model for *16S rRNA*. tvBOT (https://www.chiplot.online/tvbot.html; Xie et al. 2023) was utilised to visualise and edit the phylogenetic trees for both *COI* and *16S rRNA* sequences.

For each gene fragment, we calculated the genetic distances between sequences and elucidated the genealogical relationships among haplotypes (Xu et al. 2018; Xi et al. 2023). The genetic distances, representing the number of base substitutions, were computed using the MEGA v11.0.13 software (Tamura et al. 2021) and were estimated based on the Kimura 2‐parameter model (K2P), which was set to use 1000 bootstrap replicates and uniform rates among sites (Kimura 1980). The genealogical relationships among haplotypes were determined by constructing TCS haplotype networks utilising POPART v.1.7 (Leigh et al. 2015).

### 2.3 Morphological Analysis

Each specimen was examined for overall morphological features with the naked eye and detailed characteristics using a dissecting microscope (Nikon Stereomicroscope SMZ1270) for up‐close features. Photographs were taken with a Canon EOS 5D Mark IV camera and a Nikon Stereomicroscope SMZ1270 Imaging System. Description of anatomical parts adhered to the terminology defined by Osawa and Chan (2010). Specific morphological features were illustrated using Inkscape v1.3.2 (Inkscape 2023).

We used ImageJ v1.52a (Abràmoff et al. 2004) to measure and calculate two morphometric ratios (Kao et al. 2023) from specimen images. The first ratio is carapace length (*CL*) divided by the carapace width (*CW*), and the second is trilobate rostrum extension (*TRE*) divided by trilobate rostrum width (*TRW*), as illustrated in Figure S2. Additionally, five morphometric ratios (cheliped palm length/width, cheliped dactylus length/palm length, cheliped carpus length/width, ambulatory legs merus length/width and ambulatory legs propodus length/width) were measured indirectly from the illustrated figures. We also included images of *P. haigae* and a suspected mislabelled porcelain crab from other sources in our analysis (Miyake 1943; Sankarankutty 1963; Nakasone and Miyake 1972; Werding and Hiller 2007; online image: Chan and Lin 2013; online image: Ryanskiy, n.d.). For images without a scale bar, measurements were taken in pixels, but the ratios were compared in a similar way. All ratios were analysed using ANOVA with Tukey's post hoc tests to assess significant differences among taxa groups.

## 3 Results

### 3.1 Molecular Analyses

**FIGURE 3:**
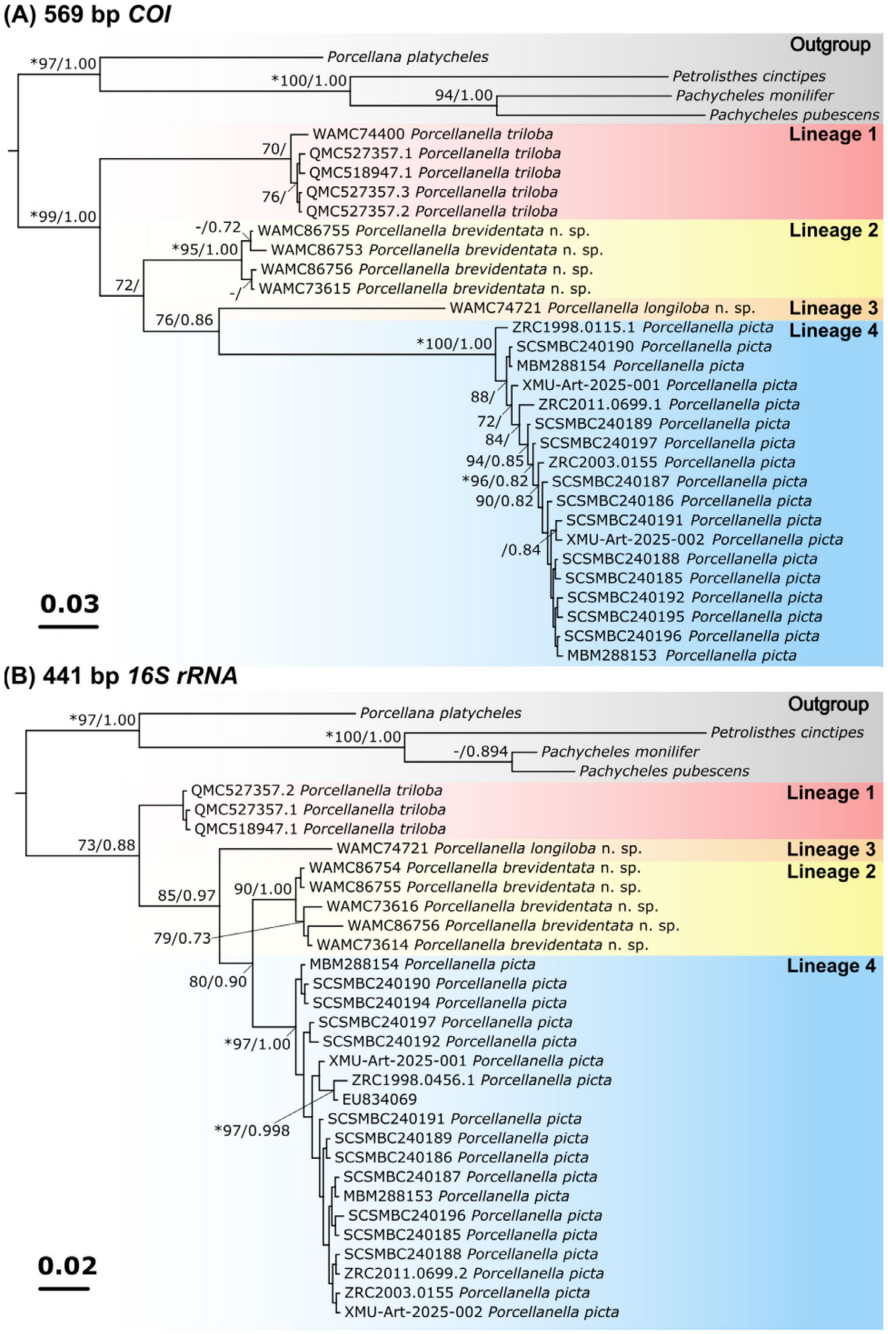
Phylogenetic trees generated by Bayesian Inference (BI) analyses for the (A) 569 bp *COI* and (B) 441 bp *16S rRNA* gene sequences of the *Porcellanella* of this study and outgroup. Values of robustness were calculated from Maximum Likelihood (ML) and BI analyses. Only bootstrap (BS) values ≥ 70 and Bayesian posterior probabilities (BPP) values ≥ 0.7 are shown at nodes. Nodes with SH‐aLRT > 80% and ultrafast bootstrap > 95% are indicated with asterisks ‘*’. The symbol ‘−’ indicates node absent in ML. GenBank accession numbers of the sequences used are listed in Table 2. The scale bar indicates the number of substitutions per site. Labelling of the Porcellanidae members followed Osawa and McLaughlin (2010).

Alignment and trimming of the amplified sequences resulted in 569 bp of *COI* and 441 bp of *16S rRNA* sequences. Our phylogenetic analyses revealed four lineages of porcelain crabs among our specimens, consistent across both *COI* and *16S rRNA* sequences (Figure 3). Australian specimens formed three lineages: lineage 1 is identified as *P. triloba* , lineage 2 is described as *P. brevidentata* n. sp. (see section 3.4), and lineage 3 is described as *P. longiloba* n. sp. (see section 3.5). All specimens in lineage 4 are from the Asia region (Hong Kong, Xiamen, Taiwan, Singapore and Thailand) and are identified as *P. picta* . Support for the four lineages as a monophyletic clade is higher in the analyses using the *COI* sequences (BS = 99, BPP = 1.00, Figure 3A) than in the *16S rRNA* sequences (BS = 73, BPP = 0.88, Figure 3B). *Porcellanella triloba* is recovered as sister to the remaining species in the analyses of both loci. The key difference between the two phylogenetic trees of each locus is that the *16S rRNA* tree recovered *P. longiloba* n. sp. as sister to a clade containing *P. brevidentata* n. sp. and *P. picta* (BS = 85, BPP = 0.97), whereas the *COI* tree recovered *P. brevidentata* n. sp. as sister to a clade containing *P. longiloba* n. sp. and *P. picta* but with much lower support (BS = 72, BPP = < 0.7). Similarly, the support for *P. brevidentata* n. sp. being sister to *P. picta* in the *16S rRNA* analyses is stronger (BS = 80, BPP = 0.90) than the support for *P. longiloba* n. sp. being sister to *P. picta* in the *COI* analyses (BS = 76, BPP = 0.86). However, none of the nodes in the above‐mentioned key differences simultaneously showed high support values in both methods: SH‐aLRT (> 80%) and ultrafast bootstrap (> 95%) (Guindon et al. 2010; Minh et al. 2013); thus, further study is required to ascertain the phylogenetic relationships of these porcellanids (see Figure S3 for depiction of additional SH‐aLRT and ultrafast bootstrap support values).

The K2P model analyses assessed the species distinctions among the four *Porcellanella* lineages using the *COI* and the *16S rRNA* markers (Table 3). The interspecific genetic divergences between the four *Porcellanella* species were 13.78% to 20.05% for *COI* and 4.41% to 8.20% for *16S rRNA*, consistently higher than the intraspecific genetic divergences of the available three *Porcellanella* species (0.49%–1.32% for *COI* and 0%–0.74% for *16S rRNA*). For pairwise comparisons between individual *Porcellanella* specimens, refer to Figure S4.

**TABLE 3:** Pairwise distance (%) based on the Kimura‐2‐parameter (K2P) model among four species of the ‘*Porcellanella triloba*’ species complex in a dataset of 569 bp *COI* sequences and 441 bp *16S rRNA* sequences generated in this work. One *16S rRNA* sequence (EU834069) from Ahyong et al. (2009) is included for the *P. picta* group. Numbers in parentheses are sample sizes. Texts of sample sizes and distance values are bolded for *COI* sequences and underlined for *16S rRNA* sequences. GenBank numbers see Table 2.

	*Porcellanella triloba*	*Porcellanella picta*	*Porcellanella brevidentata* n. sp.	*Porcellanella longiloba* n. sp.
	(3)	(19)	(5)	(1)
*Porcellanella triloba* (5)	**0.49**, 0.00	7.14	7.63	8.20
*Porcellanella picta* (18)	**20.05**	**0.93**, 0.40	4.41	7.28
*Porcellanella brevidentata* n. sp. (4)	**13.78**	**17.57**	**1.32**, 0.74	7.07
*Porcellanella longiloba* n. sp. (1)	**18.63**	**18.55**	**17.21**	N/A

**FIGURE 4:**
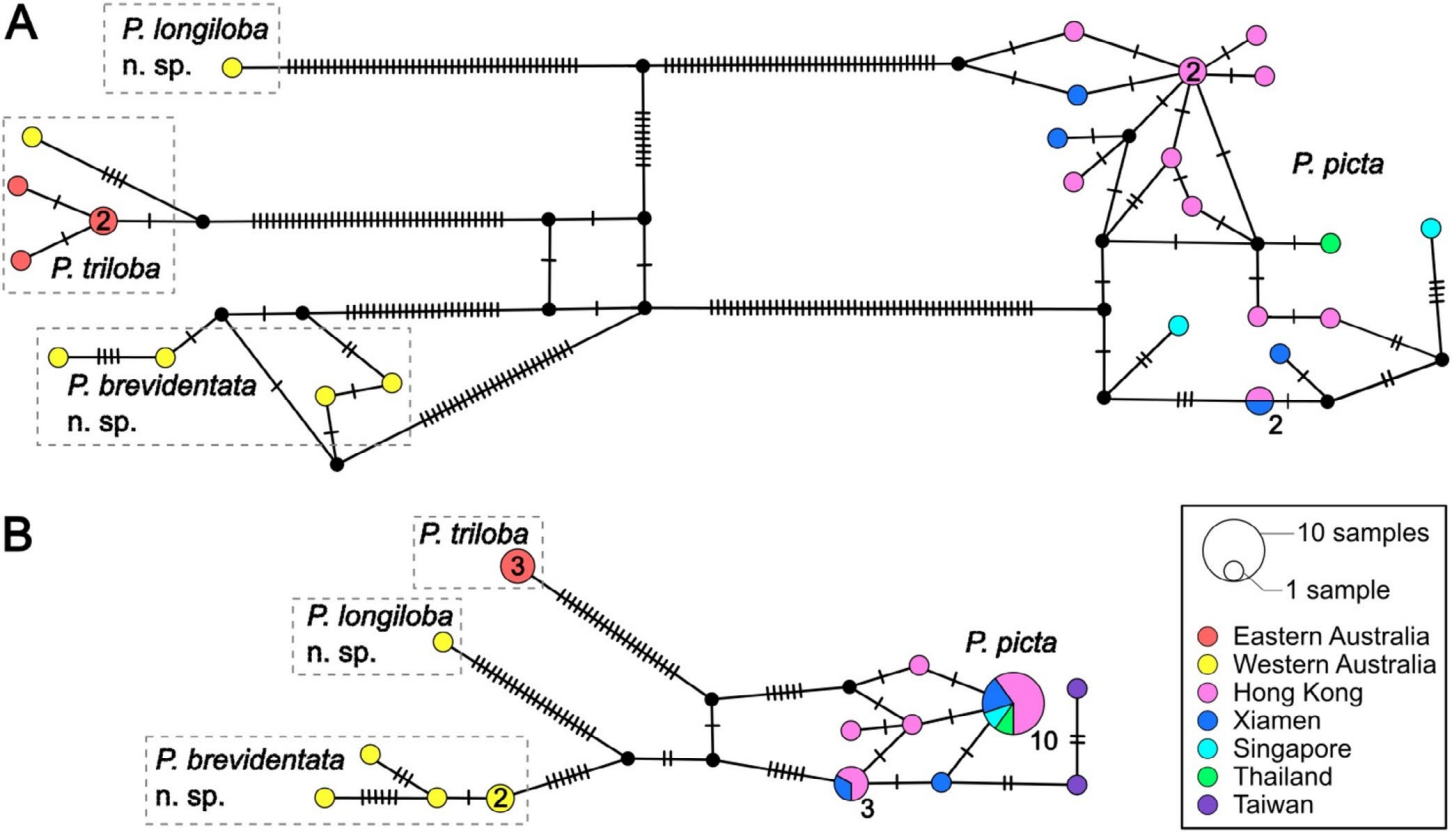
TCS network inferred based on (A) 569 bp *COI* and (B) 441 bp *16S rRNA* gene markers for haplotype of *Porcellanella triloba*, *P. picta* , *P. brevidentata* n. sp. and *P. longiloba* n. sp. in this study. Each circle represents a single haplotype. The sizes of the circles are proportional to the haplotype frequency, and where samples > 1 these are also represented by numbers. Colour of circles represent the locality of the haplotypes. Black circles indicate unknown or missing haplotypes. The hatch marks on each link indicate the number of nucleotide substitution between haplotypes. Dash lines separate the species groups of the Australian *Porcellanella* haplotypes.

The TCS haplotype networks based on *COI* (Figure 4A) and *16S rRNA* (Figure 4B) sequences illustrated the genealogical relationships among the four *Porcellanella* species from various localities, including one *16S rRNA* sequence (EU834069) obtained from GenBank (Table 2). Both markers consistently indicated similar network topology, with each species group highly segregated from the others and without any shared haplotypes. However, the *COI* network was further divided by even more haplotypes into disconnected subgroups, which consisted mostly of low‐frequency private haplotypes (Figure 4A). In contrast, the *16S rRNA* network was simpler, with the haplotype members connecting each other within each species group (Figure 4B). All Asian localities except Taiwan shared a dominant haplotype in the *16S rRNA* network, whereas in both networks, no shared haplotypes existed between the east and west of Australia.

### 3.2 Systematics

Order: Decapoda

Family: Porcellanidae Haworth, 1825

Genus: *Porcellanella* White, 1851

Type species: *Porcellanella triloba* White, 1851

Diagnosis: Carapace longer than broad; dorsal surface slightly convex and semi‐smooth; regions obscurely defined. Rostrum nearly horizontal, prominent with three flattened lobes (trilobate); median lobe exceeding lateral lobes. External orbital angle not or slightly produced. Branchial margin without spine (unarmed). Pterygostomian flaps entire. Antennal peduncle with flexible (second to fourth) articles excluded from orbit by projection of non‐flexible (first) article; first article adpressed to anterior margin of carapace. Chelipeds subequal; dactylus open obliquely. Ambulatory legs (walking legs) short with sparse setae; dactyli compressed, each with quadriunguiculate claws. Telson with 7 plates.

Remarks: Previously, this genus included only two recognised species: *P. triloba* White 1851, which our study shows is restricted to Australia, and *P. haigae* Sankarankutty, 1963, which others recorded in the Indo‐West Pacific. In this study, we resurrect *P. picta* Stimpson, 1858, which is found in the Indo‐West Pacific, and describe *P. brevidentata* n. sp. and *P. longiloba* n. sp., both occurring in Australia. All these species inhabit the leaves of Pennatulacea (sea pens), such as *Cavernularia* Valenciennes in Milne Edwards & Haime, 1850, *Pteroeides* Herklots, 1858, *Veretillum* Cuvier, 1798 and *Virgularia* Lamarck, 1816, on muddy or sandy bottoms. During our morphological examination, only *P. picta* specimens were freshly collected and exhibit clear markings and plumose setae. The remaining species ( *P. triloba* , *P. brevidentata* n. sp. and *P. longiloba* n. sp.) were examined from preserved museum specimens, some of which had faded markings and hardened setae.

### 3.3 *Porcellanella triloba* White, (1851)

**FIGURE 5:**
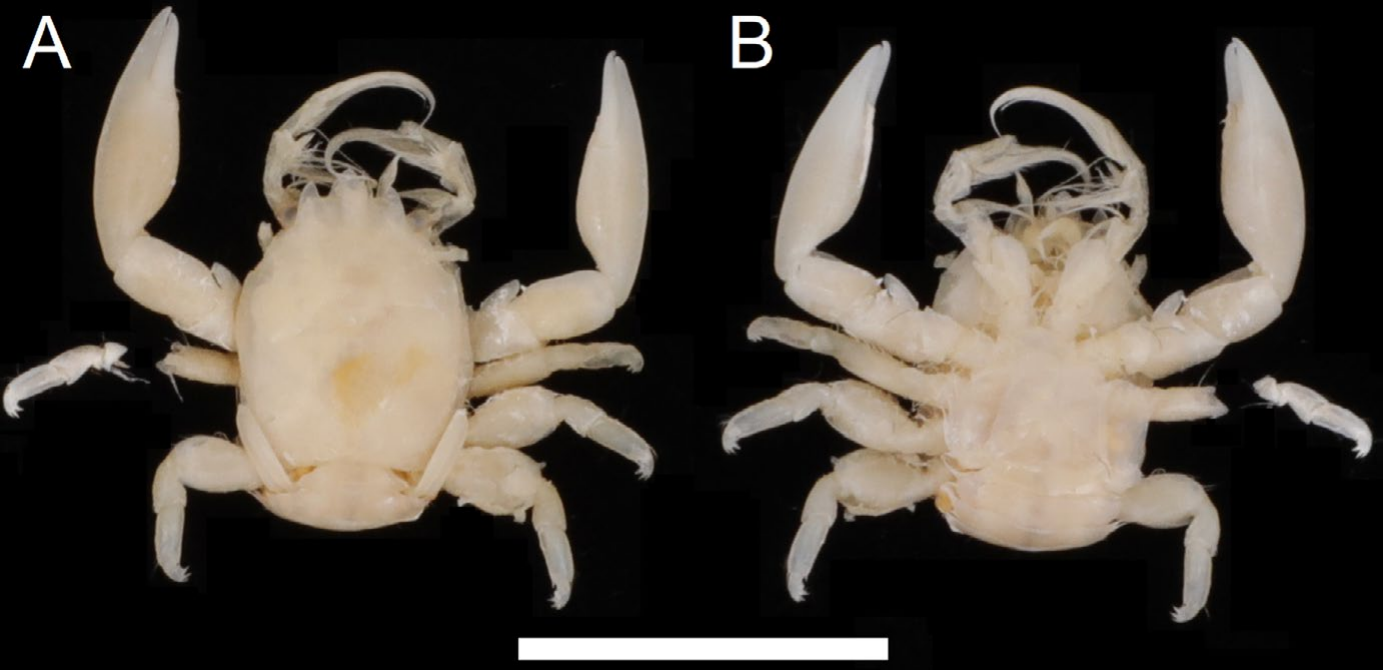
Dorsal (A) and ventral (B) view of *Porcellanella triloba* White, museum collection specimen QMC518947.1 (neotype). Scale bar = 10 mm.

See Figures 5, 9, 13A.

*Porcellanella triloba*: White, (1851), 394–395, figure 2, 2a [**type locality:** off Cape Capricorn, Queensland]—Johnson, (1964), 98–102.

Not *Porcellanella triloba*: Henderson, 1893, 429—Lens, 1905, 341–392—Barnard, 1950, 819–821—Macnae and Kalk 1958, 126—Sankarankutty, 1961, 96–100, figure 1—Haig, 1992, 323–324, figure 19. —Ahyong et al. 2009, 14, table 1—Osawa and Chan 2010, 175–177, figures 138 and 139—Chowdhury and Mitra 2023, 133–135, figures 1B–D and 2D–f. [= *Porcellanella picta* Stimpson, 1858; synonymy resurrection].

? *Porcellanella triloba*: Henderson, 1893, 429 (plausible mislabelled)—Miyake, 1943, 134, figure 53 (depicting sketch shows cheliped without meral lobe). [not *P. triloba* White, 1851].

Type material: Neotype: **Australia** • QMC518947.1 (Figure 5), collected on 19 September 2004 from the Great Barrier Reef, Queensland (22°44′42.0″S, 150°51′18.0″ E).

Additional specimens examined: **Australia** • QMC518947.2, collected on 19 September 2004 from the Great Barrier Reef, Queensland (22°44′42.0″S, 150°51′18.0″ E); QMC527357.1–3, collected on 10 November 2005 from the Great Barrier Reef, Queensland (22°42′54.0″S, 150°57′54.0″ E); WAMC74400, collected on 25 February 2019 from Shark Bay, Western Australia (24°56′01.0″S, 113°23′34.0″ E).

Description: Carapace elongated, with average *CL*/*CW* ratio 1.34 (*n* = 6, Table S5). Rostrum protruding with three lobes and average *TRE*/*TRW* ratio 0.43 (*n* = 6, Table S5); median lobe triangular with subacute tip; lateral lobes curved inwards with convex lateral margins. External orbital angles acute and slightly produced (Figures 9A and 13A1). Pterygostomian flap with spinule on upper margin posterior to antennal peduncle (Figures 9B and 13A2).

Third thoracic sternite attaching to third maxilliped thick, central process semi‐rounded with anterior margins broadly triangular, much larger than lateral lobes (Figure 9C).

Basal article of antennular peduncle longer than broad; anterior and lateral margins furnished with setae; ventral surface striated with setae; anterior plate much prolonged, with 2 acute spinules at mesial angles (Figures 9D and 13A3).

Antennal peduncle unarmed, basal article ventrally covered by pterygostomian flap (Figures 9B and 13A2).

Third maxilliped striated, bearing long plumose setae on mesial margins of carpus, propodus and dactylus (Figure 5B); ischium produced distally on extensor margin; merus as long as ischium, extensor margin straight, and flexor margin with subrectangular lobe; carpus triangular, divergent distally; propodus subequal with carpus in length (Figure 9E).

Chelipeds subequal; rather slender; surface smooth and glossy like carapace. Palm elongated, 2.94 times longer than broad, with line of dense plumose setae ventrally; dactylus to palm ratio 0.37. Carpi relatively short, 1.45 times longer than broad. Meri with distinct subtriangular lobe with semi‐rounded tip on dorsoflexor margin distally (Figures 9F and 13A4).

Ambulatory legs small, furnished with setae; merus longer than broad at 1.86, 1.64 and 1.30 ratios respectively for 2nd to 4th pereopods (Figure 9G–I); propodus longer than broad at 2.39, 2.50 and 2.34 ratios respectively for 2nd–4th pereopods (Figure 9G–I), with a pair of spinules at the distal end of the posterior margin; dactylus armed with four sharp unguicles (claws): second and third subequal, fourth smallest and about half of first (Figures 9J and 13A5).

Coloration in preservation: Overall body pale yellowish or white (Figure 5).

Distribution: Known with certainty from Australia: Cape Capricorn (type locality), Bowen and off Great Barrier Reef in Queensland; and also from Shark Bay in Western Australia.

Habitat: The specimen WAMC74400 was collected from a water depth of 21.5 m.

Remarks: The most distinct feature of *P. triloba* is the presence of a spinule at the upper margin of the pterygostomian flap near the posterior of the antennal peduncle (referred as ‘ventral margin of the antennal groove with a prominent spinule in the middle of its length’ in Johnson 1964), therefore we selected this phenotype as *P. triloba* among the three Australian lineages revealed from our phylogenetic analyses (Figure 13A2). Whereas the other three species in this study (*P. brevidentata* n. sp., *P. longiloba* n. sp., and *P. picta* ) and *P. haigae* (online image: Charpin, n.d.) lack this feature. In *P. triloba* , the cheliped merus has a meral lobe (Figure 13A4), which differentiates it from *P. longiloba* n. sp. (Figure 13D4) and *P. haigae* (Figure S6; Sankarankutty, 1963: figure 1C) that lack this feature. The fourth distal unguicles on its ambulatory legs are very small, like half or less than half, the size of the first distal unguicle (Figure 13A5), which differentiates it from *P. picta* 's larger ones that are subequal to the first distal unguicle (Figure 13B5).

*Porcellanella triloba* was initially described from a single specimen collected off Cape Capricorn (23**°** 25′S, 151**°** 12′ E; White 1851). Although White's description of this holotype lacks the distinct details to differentiate with other Australian *Porcellanella* species, the sketch image depiction (White 1851: figure 2) has a meral lobe at chelipeds (further excluding *P. longiloba* n. sp.) and the median rostral lobe is triangular with a semi‐acute tip (further excluding *P. brevidentata* n. sp.), which matches our *P. triloba* specimens (WAMC74400, QMC518947.1–2, QMC527357.1–3). Although Henderson, (1893) had examined White's type of *P. triloba* at the Natural History Museum, London (=British Museum), this type (catalogue no. 1888.33) cannot be located (Paul Clark, Miranda Lowe, personal communications). As our study revealed multiple species from the *P. triloba* species complex with subtle differences, we consider it necessary to elect a neotype for *P. triloba* . Specimen QMC518947.1 is herein selected to be the neotype of *P. triloba* because of its relatively good condition (only two detached legs, Figure 5), relatively large size (*CL* = 8.12 mm), available genetic sequences on the GenBank database (Table 2), and collected from the Great Barrier Reef (22°44′42.0″S, 150°51′18.0″ E) that is relatively close to the type locality off Cape Capricorn.

Previously, *P. triloba* was thought to be the most widely distributed *Porcellanella*. The specimen descriptions and figure depictions of ‘ *P. triloba* ’ in Henderson, (1893), Sankarankutty, (1961) and Chowdhury and Mitra (2023) are consistent with the *P. picta* in our work (e.g., the fourth distal unguicle, or the first proximal unguicle, was large or subequal to the other unguicles; Figures 10J and 13B5). Therefore, we suggest that the distribution locations of the Celebes Sea, the Gulf of Mannar and the Hooghly River mouth of West Bengal belong to *P. picta* .

The ‘*Porcellanella triloba*’ in Miyake (1942, 1943) not only lacks a cheliped meral lobe (conflicting with *P. triloba* morphology) but also has exceptionally longer *CL*/*CW* and *TRE*/*TRW* ratios than *P. triloba* , see Table S5. Additionally, the basal article of antennular peduncle depicted in Miyake 1942: figure 29a has 5 spinules, which is different from *P. haigae* 's that has 4 spinules (Sankarankutty 1963: figure 1b) and *P. longiloba* n. sp.'s that has 3 spinules (Figures 12D and 13D3). Therefore, we considered this specimen of Miyake (1943) not *P. triloba* and excluded Palau Island from its distribution.

Johnson (1964) proposed that records of *P. triloba* from eastern Africa (Lens 1905; Barnard 1950; Macnae and Kalk 1958) should be reassigned to *P. picta* , as earlier studies had treated the distinguishing features of *P. triloba* as mere variants of *P. picta* . However, given the long distance between the localities of the East African records and the confirmed distribution of this species in Australia, we suggest that further studies should be conducted to determine whether these records represent another cryptic species of *Porcellanella*.

Although *P. triloba* was also recorded in the Falkland Islands in the Southern Atlantic by Henderson (1893), given that Haig (1981) considered this record to be the result of a labelling error, we disregard this as a valid record of *P. triloba.*

Our examination of ‘*Porcellanella triloba*’ depictions of the Hong Kong specimen in Haig (1992: figure 19C) and the Taiwan specimen in Osawa and Chan (2010: figure 138) revealed that the fourth distal unguicle on the ambulatory legs is subequal in size to the other unguicles. Based on this characteristic, we considered these works were actually depicting *P. picta* instead of *P. triloba* .

Although Ahyong et al. (2009) did not include any description or figure depiction for their Taiwan ‘*Porcellanella triloba*’ specimen of *16S rRNA* sequence (GenBank: EU834069), we included the EU834069 sequence in our molecular analyses and revealed that it belongs to *P. picta* (Figures 3B, 4B; Figure S4B).

Overall, our study shows that the distribution of *P. triloba* is confined to Australia (White 1851; Johnson 1964; this work) and previous records of this species in Asia should be *P. picta* .

### 3.4 *Porcellanella picta* Stimpson, 1858. (Resurrected Species Name)

**FIGURE 6:**
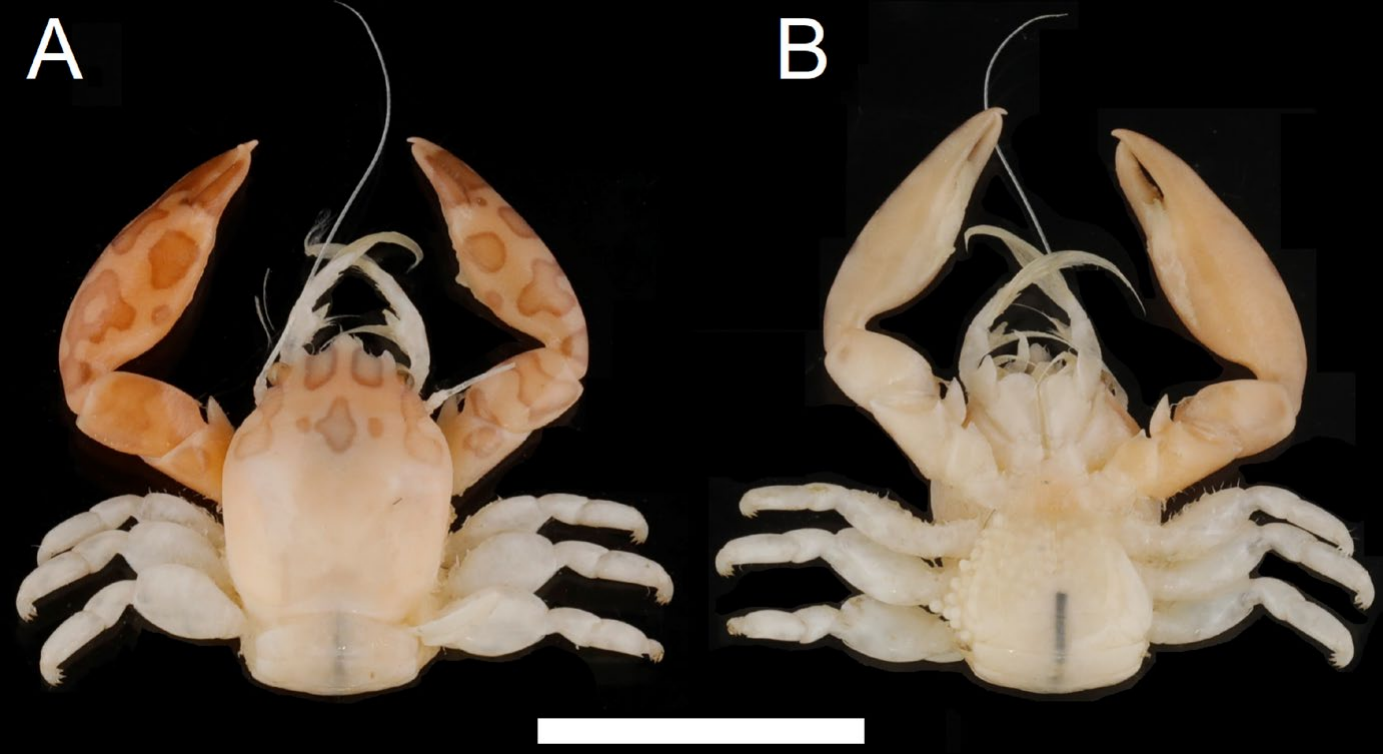
Dorsal (A) and ventral (B) view of *Porcellanella picta* Stimpson, fieldwork collection specimen SCSMBC240192 (neotype). Scale bar = 10 mm.

See Figures 2, 6, 10, 13B; Figure S7.

*Porcellanella picta*: Stimpson, 1858, 243–244; Stimpson, 1907, 193, Pl. 22, figure 6 [**type locality:** Hong Kong]—de Man, 1888, 220–221—Yokoya, 1933, 70—Miyake, 1943, 134–137, figures 54 and 55—Johnson, 1964, 98–102, figure 1—Sivasubramanian et al. 2014, 248–251, figures 2 and 3.

*Porcellanella triloba*: Henderson, 1893, 429—Lens, 1905, 341–392—Barnard, 1950, 819–821—Macnae and Kalk 1958, 126—Sankarankutty, 1961, 96–100, figure 1—Haig, 1992, 323–324, figure 19—Ahyong et al. 2009, 14, table 1—Osawa and Chan 2010, 175–177, figures 138 and 139—Chowdhury and Mitra 2023, 133–135, figures 1B–D and 2D–f. [= *Porcellanella picta* Stimpson, 1858; synonymy resurrection].

Type material: Neotype: **Hong Kong** • SCSMBC240192 (Figure 6), collected on 22 March 2023 from Kau Yi Chau South (22°16′18.8″N, 114°04′44.9″ E).

Additional specimens examined: **Hong Kong** • SCSMBC240195, collected on 22 March 2023 from Kau Yi Chau South (22°16′18.8″N, 114°04′44.9″ E); SCSMBC240190–240191, collected on 10 June 2022 from Hei Ling Chau (22°15′31.7″N, 114°02′26.4″ E); SCSMBC240185–240188 and SCSMBC240197, collected on 13 April 2022 and SCSMBC240193–240194, collected on 22 March 2023 from Lamma Off (22°10′06.1″N, 114°10′55.9″ E); SCSMBC240189, collected on 25 April 2022 from Lamma East (22°11′21.4″N, 114°10′05.2″ E); SCSMBC240198–240200, collected on 12 July 2022 from Shek Kwu Chau (22°11′41.2″N, 114°00′13.8″ E). **Singapore** • ZRC1998.0115.1–2, collected on 12 March 1998 from Changi Beach (coordinates n.d.); ZRC2011.0699.1–2, collected on August 2011 from Pulau Tekong (coordinates n.d.); ZRC1998.0113, collected on 12 March 1998 from Bedok Jetty (coordinates n.d.). **Thailand** • ZRC2003.0155, collected on 6 June 2003 from Pattani fish port (coordinates n.d.); ZRC2000.0907.1, collected on 20 February 2000 from Angsila fish port (coordinates n.d.). **Taiwan** • ZRC1998.0456.1, collected on 25 May 1998 from fish port at Tashi (coordinates n.d.). **Xiamen** • MBM288153–288154 and XMU‐Art‐2025‐001–002, collected on 2 July 2022 from Shuangyu Island East (24°22′56.1″N, 118°06′01.0″ E).

Description: Carapace elongated with average *CL*/*CW* ratio 1.26 (*n* = 15, Table S5). Rostrum protruding with three lobes and average *TRE*/*TRW* ratio 0.33 (*n* = 15, Table S5); median lobe broadly triangular with acute tip; lateral lobes curved inwards with lateral margins more convex. External orbital angles rounded and not produced (Figures 10A and 13B1). Pterygostomian flaps unarmed on upper margin (Figures 10B and 13B2).

Third thoracic sternite attaching to third maxilliped thick, central process with anterior margins rounded, much larger than lateral lobes (Figure 10C).

Basal article of antennular peduncle longer than broad; anterior and lateral margins furnished with setae; ventral surface striated with setae; anterior plate much prolonged, with 2 acute spinules at mesial angles (Figures 10D and 13B3).

Antennal peduncle unarmed, basal article ventrally covered by pterygostomian flap (Figures 10B and 13B2).

Third maxilliped striated, bearing long plumose setae on mesial margins of carpus, propodus and dactylus (Figure 6B); ischium produced distally on extensor margin; merus as long as ischium, extensor margin straight, and flexor margin with subrectangular lobe; carpus triangular, divergent distally; propodus subequal with carpus in length (Figure 10E).

Chelipeds subequal; rather slender; surface smooth and glossy like carapace. Palm elongated, 3.39 times longer than broad, with line of dense plumose setae ventrally; dactylus to palm ratio 0.36. Carpi relatively short, 1.41 times longer than broad. Meri with a distinct subtriangular lobe with semi‐acute tip on dorsoflexor margin distally (Figures 10F and 13B4).

Ambulatory legs small, furnished with setae; merus longer than broad at 1.65, 1.75 and 1.63 ratios respectively for 2nd to 4th pereopods (Figure 10G–I); propodus longer than broad at 2.51, 2.35 and 2.71 ratios respectively for 2nd–4th pereopods (Figure 10G–I), with a pair of spinules at the distal end of the posterior margin; dactylus armed with four sharp unguicles: third slightly larger than second, fourth and first subequal in size but relatively shorter than second and third (Figures 10J and 13B5).

Coloration in life: Overall body of the live specimen glossy white underwater (Figure 2A), yellowish‐white when fresh out of the water (Figure 2B; Figure S7). Dark pinkish brown markings, in the form of oval to irregularly‐shaped patches with the edges in darker colour, are present on dorsal chelipeds and anterior carapace (Figures 2 and 6A; Figure S7A–C); no observable pigment markings are present on the ventral side (Figure 6B; Figure S7D).

Coloration in preservation: Overall body of the preserved specimen is pale yellowish or white, and the colour markings gradually fade after prolonged ethanol preservation (Figure 6). Nevertheless, some specimens preserved for more than 10 years may still show observable colour markings.

Distribution: This study confirmed the occurrence of *P. picta* in eastern Asia. Its range extends from the southern waters of Hong Kong (type locality) to Xiamen, Taiwan, the Gulf of Thailand, and Singapore. Localities from past studies include Japan, Celebes Sea Andaman Sea, Southeastern India and eastern Africa.

Habitat: Specimens collected in Hong Kong at 10 to 35 depths (SCSMBC240185–240200) were found residing in the leaves of their sea pen hosts, either *Pteroeides* or *Virgularia*.

Remarks: The most distinct features of *P. picta* are the colour markings on the chelipeds and the carapace, and the relatively large size of the fourth distal unguicle on the dactylus of the ambulatory leg, which is subequal to the first distal unguicle (Johnson 1964), whereas the fourth distal unguicle in the other species is half, or less than half, the size of the first distal unguicle (Figure 13A5,B5,C5,D5). In addition, the merus of the cheliped of *P. picta* has a meral lobe (Figure 13B4), which differentiates it from *P. longiloba* n. sp. (Figure 13D4) and *P. haigae* (Figure S6), which lack this feature.

*Porcellanella picta* was described from a single male specimen collected at Hong Kong port (Stimpson 1858). Stimpson's description is relatively general and hardly able to differentiate it from the other cryptic Australian species. Nevertheless, our phylogenetic work indicates that *Porcellanella* specimens from the Asia region (Table 1) are *P. picta* (Figures 1, 3, 4). Stimpson's types were mostly lost in the Great Chicago Fire in October 1871. Although Henderson, (1893) had examined specimens, possibly including type specimens, of *P. picta* at the Natural History Museum, London, these specimens are now missing (Paul Clark, Miranda Lowe, personal communications). As our study revealed multiple *Porcellanella* species with subtle differences, it is necessary to elect a neotype for *P. picta* . Specimen SCSMBC240192 was chosen to be the neotype of *P. picta* because of its good condition (Figure 6), relatively large size (*CL* = 8.61 mm), available genetic sequences on the GenBank database (Table 2), and collected from Kau Yi Chau South (22°16′18.8″N, 114°04′44.9″ E) that is within Hong Kong Harbour, which is the original type locality.

Since we propose the resurrection of *P. picta* , previous related studies should affirm its presence. The specimen descriptions in Henderson (1893), Sankarankutty (1961) and Chowdhury and Mitra (2023) are consistent with the *P. picta* in our work (e.g., the fourth distal unguicle, or the first proximal unguicle, was large or subequal to the other unguicles; Figures 10J and 13B5). Therefore, we suggest that the distribution locations of the Celebes Sea, the Gulf of Mannar and the Hooghly River mouth of West Bengal belong to *P. picta* .

Our observation of ‘*Porcellanella triloba*’ depictions in the Hong Kong specimen of Haig (1992: figure 19C) and the Taiwan specimen of Osawa and Chan (2010: figure 138) revealed subequal size of the fourth distal unguicle with the first distal unguicle in the ambulatory legs; therefore, we considered their works were depicting *P. picta* instead of *P. triloba* .

Although Ahyong et al. (2009) did not include any description or figure depiction for their Taiwan ‘*Porcellanella triloba*’ specimen of *16S rRNA* sequence (GenBank: EU834069), we included the EU834069 sequence in our molecular analyses and revealed that it belongs to *P. picta* (Figures 3B, 4B; Figure S4B).

Overall, our study shows that *P. picta* is a species widely distributed in eastern Asia, with records from Japan to Singapore.

### 3.5 *Porcellanella brevidentata* Loke, Hosie and Qiu n. sp.

ZooBank registration LSID: urn:lsid:zoobank.org:act:11122F0E‐D7F9‐4552‐AF81‐A5C22EA2BA95.

**FIGURE 7:**
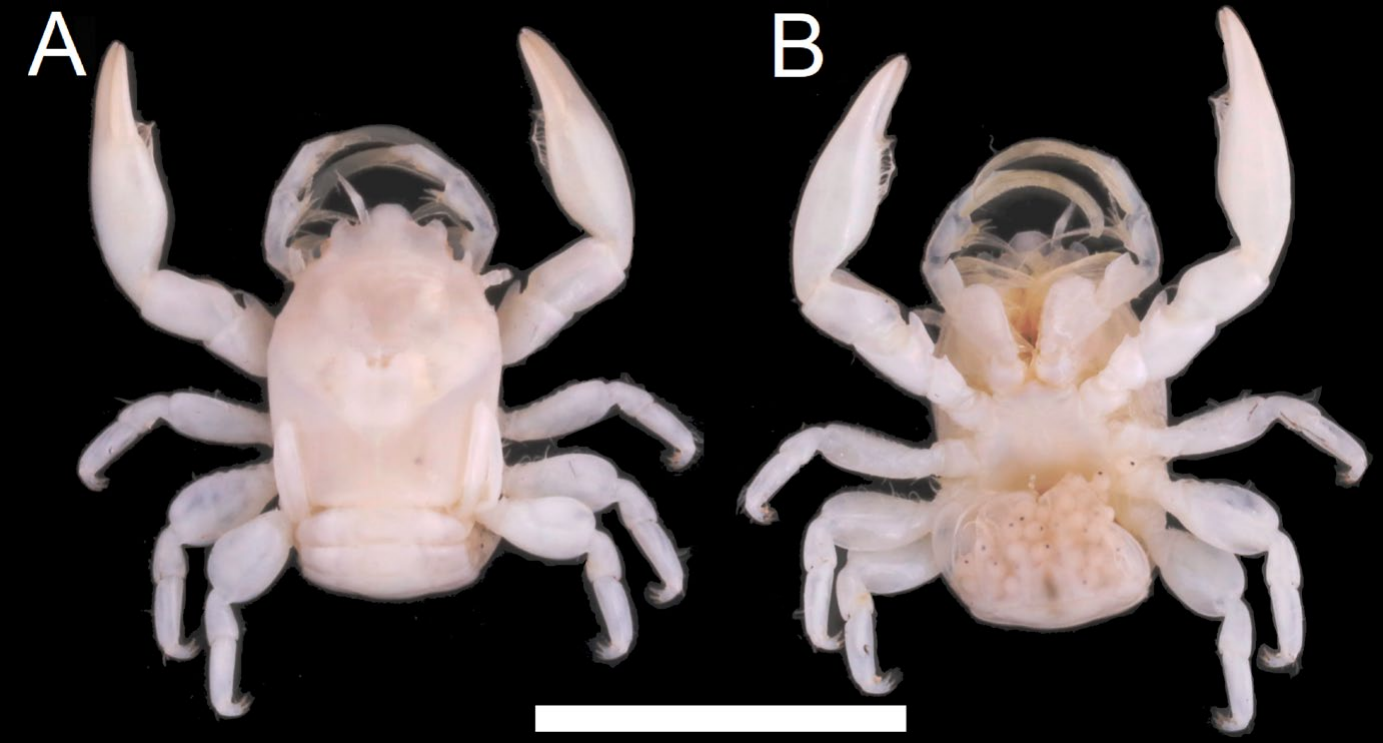
Dorsal (A) and ventral (B) view of *Porcellanella brevidentata* n. sp., museum collection specimen WAMC73614 (holotype). Scale bar = 10 mm.

See Figures 7, 11, 13C.

Type material: Holotype: **Western Australia** • WAMC73614 (Figure 7), collected on 1 November 2017 from northeast of Trimouille Island, North West Shelf (20°11′42.7″S, 115°57′26.3″ E) as the type locality. Paratypes: **Western Australia** • WAMC44990.1–2, collected on 31 January 2008 from Ningaloo Marine Park (21°48′03.7″S, 114°00′14.1″ E to 21°48′07.3″S, 114°00′16.8″ E); WAMC73615 and WAMC86753, collected on 5 November 2017 from northeast of Trimouille Island, North West Shelf (20°11′56.4″S, 115°47′13.2″ E); WAMC73616, collected on 2 November 2017 from North West Shelf (20°10′49.1″S, 115°57′00.7″ E); WAMC79506.1–2 and WAMC86754–6, collected on 13 December 2022 from Gascoyne Marine Park (23°54′25.9380″S, 113°05′51.6264″ E).

Description: Carapace elongated, with average *CL*/*CW* ratio 1.27 (*n* = 10, Table S5). Rostrum slightly protruding with three short lobes and average *TRE*/*TRW* ratio 0.34 (*n* = 10, Table S5); median lobe triangular with rounded tip; lateral lobes slightly curved inwards with lateral margins slightly convex. External orbital angles rounded and not produced (Figures 11A and 13C1). Pterygostomian flaps unarmed on upper margin (Figures 11B and 13C2).

Third thoracic sternite attaching to third maxilliped thick, central process with anterior margins semi‐triangular, much larger than lateral lobes (Figure 11C).

Basal article of antennular peduncle longer than broad; anterior and lateral margins furnished with setae; ventral surface striated with setae; anterior plate much prolonged, with 2 acute spinules at mesial angles (Figures 11D and 13C3).

Antennal peduncle unarmed, basal article ventrally covered by pterygostomian flap. (Figures 11B and 13C2).

Third maxilliped striated, bearing long plumose setae on mesial margins of carpus, propodus and dactylus (Figure 7B); ischium produced distally on extensor margin; merus as long as ischium, extensor margin straight, and flexor margin with subrectangular lobe; carpus triangular, divergent distally; propodus subequal to carpus in length (Figure 11E).

Chelipeds subequal; rather slender; surface smooth and glossy like carapace. Palm elongated, 3.12 times longer than broad, with line of dense plumose setae ventrally; dactylus to palm ratio 0.38. Carpi relatively short, 1.42 times longer than broad. Meri with a distinct subtriangular lobe with semi‐rounded tip on dorsoflexor margin distally (Figures 11F and 13C4).

Ambulatory legs small, furnished with setae; merus longer than broad at 2.00, 1.66 and 1.69 ratios respectively for 2nd to 4th pereopods (Figure 11G–I); propodus longer than broad at 2.48, 2.70 and 2.55 ratios respectively for 2nd to 4th pereopods (Figure 11G–I), with a pair of spinules at the distal end of the posterior margin; dactylus armed with four sharp unguicles: third relatively larger than second, fourth smallest and about half of first (Figures 11J and 13C5).

Coloration in preservation: Overall body pale yellowish or white (Figure 7).

Etymology: The specific name is derived from the Latin ‘*brevis*’ and ‘*dentata*’, meaning ‘short‐toothed’, alluding to the median and lateral rostral lobes of this species, which are rounded and visually shorter than those of other Australian *Porcellanella* species.

Distribution: Currently known from Trimouille Island (type locality), Gascoyne Marine Park and the North West Shelf in Western Australia.

Habitat: Specimens collected in Gascoyne Marine Park at 122 m depth (WAMC79506.1–2 and WAMC86754–6) were found residing among the leaves of their *Pteroeides* sea pen hosts. Other specimens (WAMC44990.1–2, WAMC73614–6, WAMC86753) were collected from unknown habitats at water depths 53 to 61 m.

Remarks: Among the three Australian lineages revealed from our phylogenetic analyses, this lineage with the shortest extension of the trilobate rostrum was described as *P. brevidentata* n. sp. (average *TRE*/*TRW* ratio = 0.34; ANOVA test *p* < 0.001; see Table S5 for measurement details and Tukey's comparison groupings).

The upper margin of its pterygostomian flap, posterior to the basal antennal peduncle, lacks spinules (Figure 13C2), which differentiates it from *P. triloba* (Figure 13A2). The merus of its cheliped has a meral lobe (Figure 13C4), which differentiates it from *P. longiloba* n. sp. and *P. haigae* that are both without such a meral lobe (Figure 13D4; Figure S6; Sankarankutty 1963: figure 1c). The fourth distal unguicles on its ambulatory legs are very small, like half or less than half, the size of the first distal unguicle (Figure 13C5), which differentiates it from *P. picta* 's larger ones that are subequal to the first distal unguicle (Figure 13B5).

### 3.6 *Porcellanella longiloba* Loke, Hosie and Qiu n. sp.

ZooBank registration LSID: urn:lsid:zoobank.org:act:9FEB8BCF‐CBBA‐4027‐AEA7‐8B612EA5AFB9.

**FIGURE 8:**
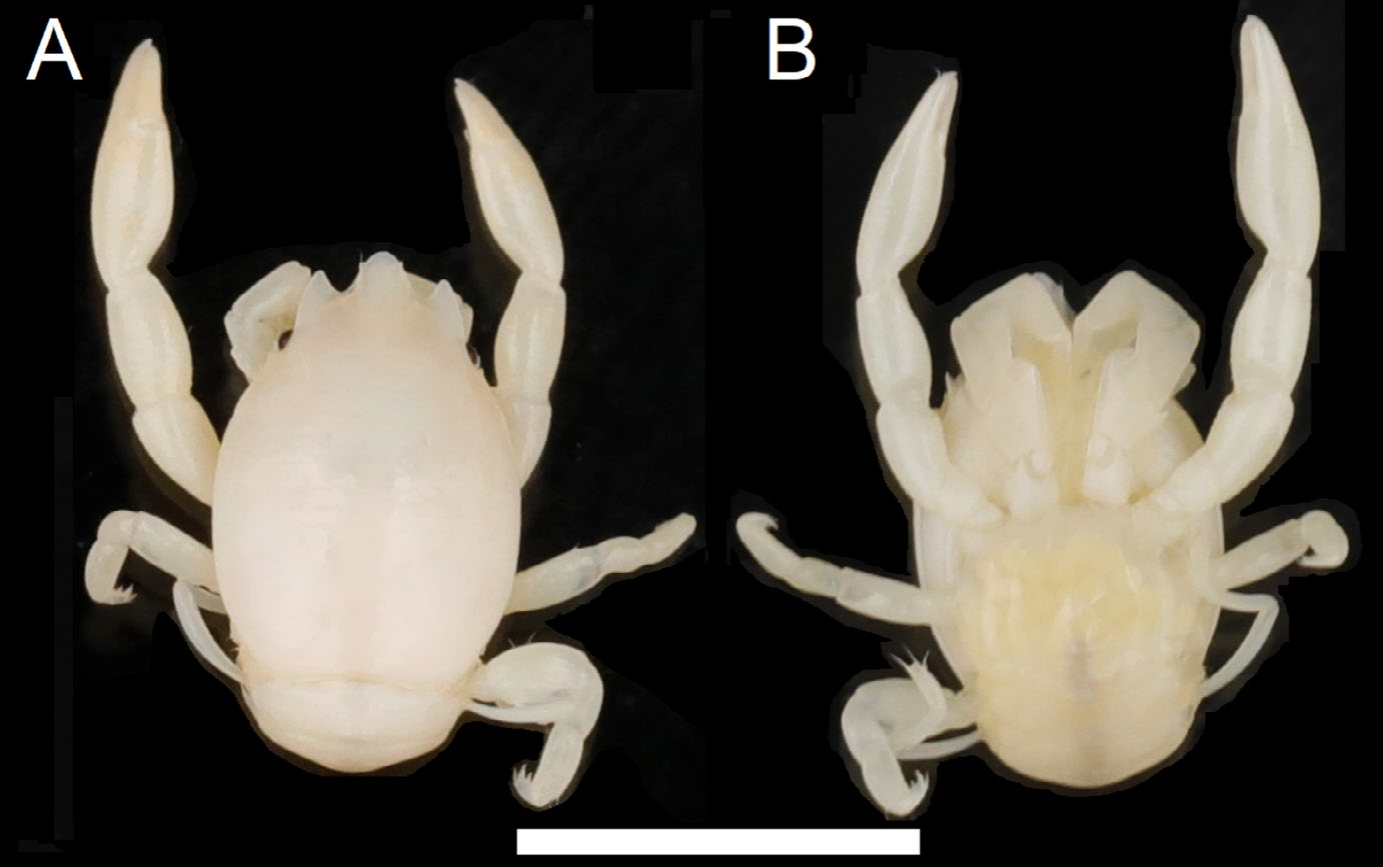
Dorsal (A) and ventral (B) view of *Porcellanella longiloba* n. sp., museum collection specimen WAMC74721 (holotype). Scale bar = 5 mm.

See Figures 8, 12, 13D; Figure S6A–B.

**FIGURE 9:**
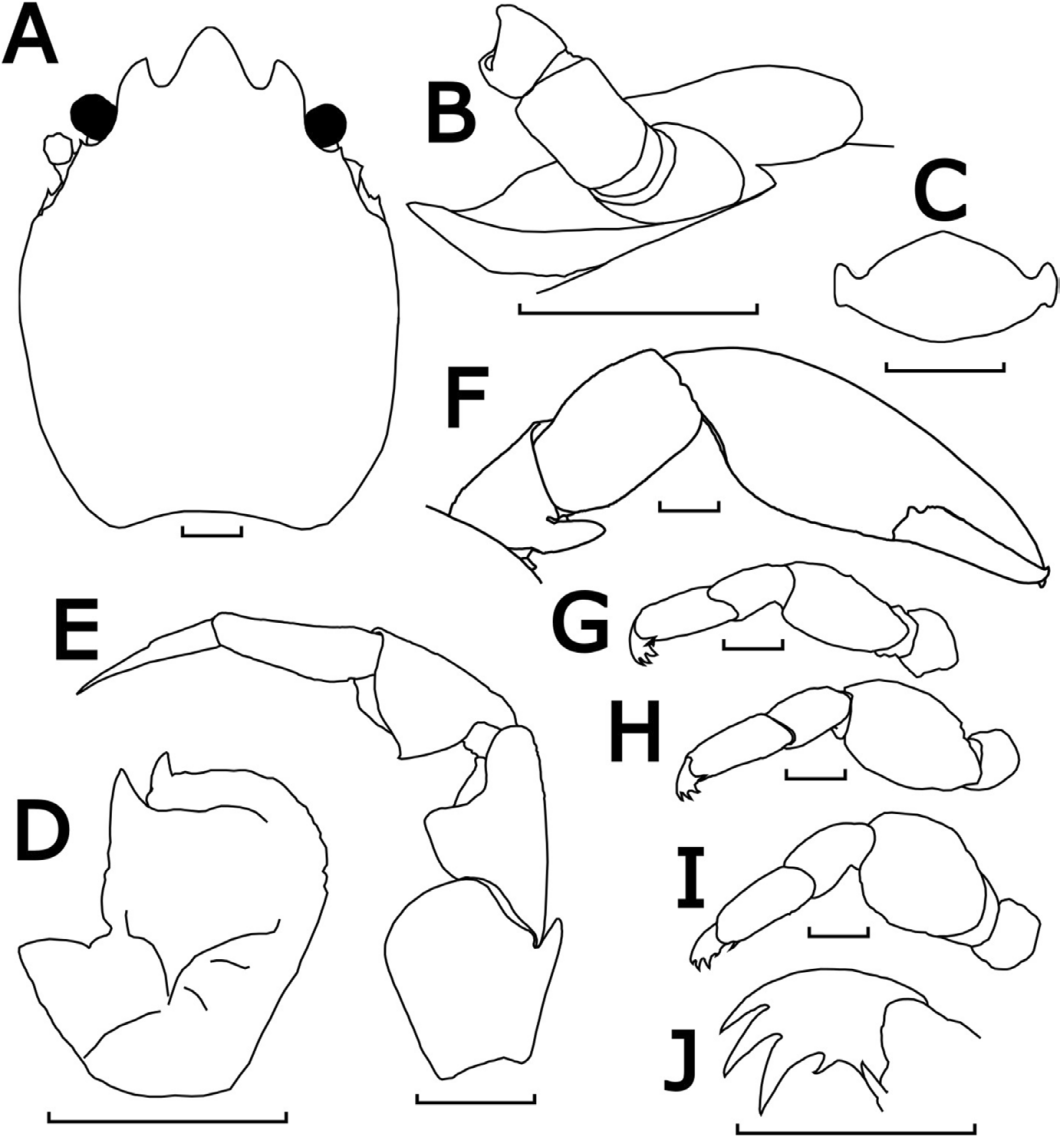
Morphological illustrations of *P. triloba* , QMC518947.1 (neotype). (A) Carapace, dorsal view; (B) upper margin of pterygostomian flap, posterior to left basal article of antennal peduncle, semi‐lateral view; (C) third thoracic sternite, ventral view; (D) left basal article of antennular peduncle, ventral view; (E) left third maxilliped, ventral view; (F) left cheliped, dorsal view; (G–I) horizontally inverted right ambulatory legs (2nd–4th pereopods), lateral view; (J) left dactylus of 4th pereopod, dorsal view. Scale bar = 1 mm.

**FIGURE 10:**
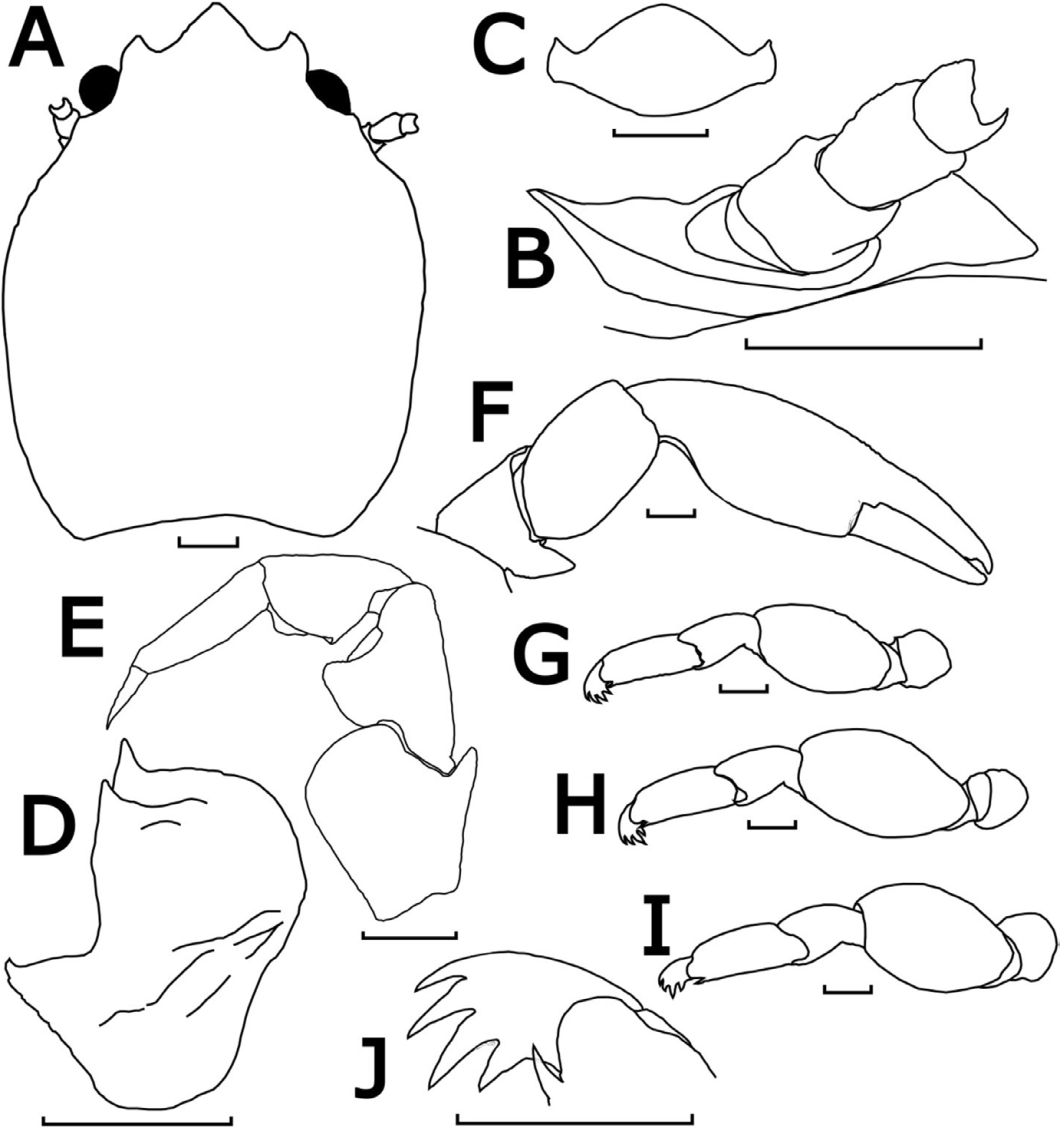
Morphological illustrations of *P. picta* , SCSMBC240192 (neotype). (A) Carapace, dorsal view; (B) upper margin of pterygostomian flap, posterior to left basal article of antennal peduncle, semi‐lateral view; (C) third thoracic sternite, ventral view; (D) left basal article of antennular peduncle, ventral view; (E) left third maxilliped, ventral view; (F) left cheliped, dorsal view; (G–I) left ambulatory legs (2nd–4th pereopods), lateral view; (J) left dactylus of 2nd pereopod, dorsal view. Scale bar = 1 mm.

**FIGURE 11:**
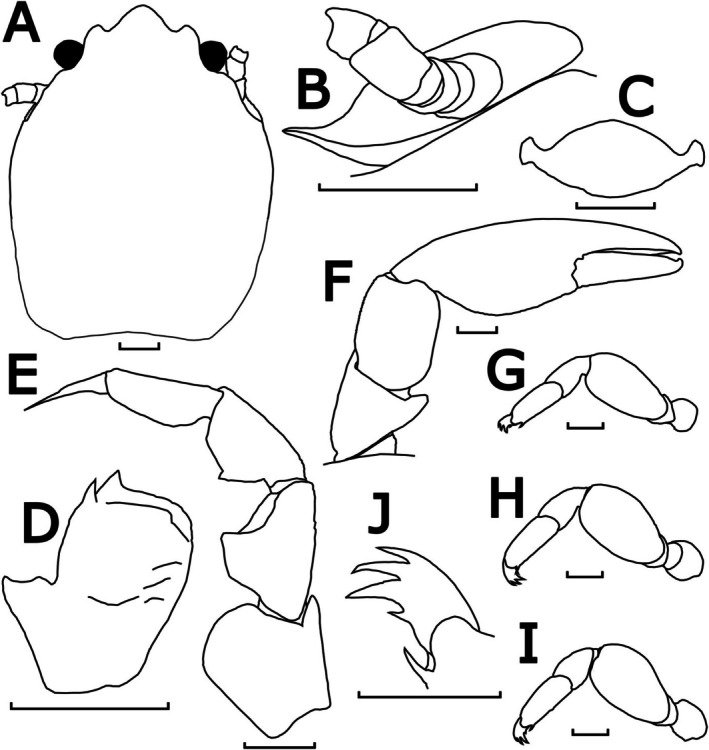
Morphological illustrations of *P. brevidentata* n. sp., WAMC73614 (holotype). (A) Carapace, dorsal view; (B) upper margin of pterygostomian flap, posterior to left basal article of antennal peduncle, semi‐lateral view; (C) third thoracic sternite, ventral view; (D) left basal article of antennular peduncle, ventral view; (E) left third maxilliped, ventral view; (F) left cheliped, dorsal view; (G–I) left ambulatory legs (2nd–4th pereopods), lateral view; (J) left dactylus of 3rd pereopod, dorsal view. Scale bar = 1 mm.

**FIGURE 12:**
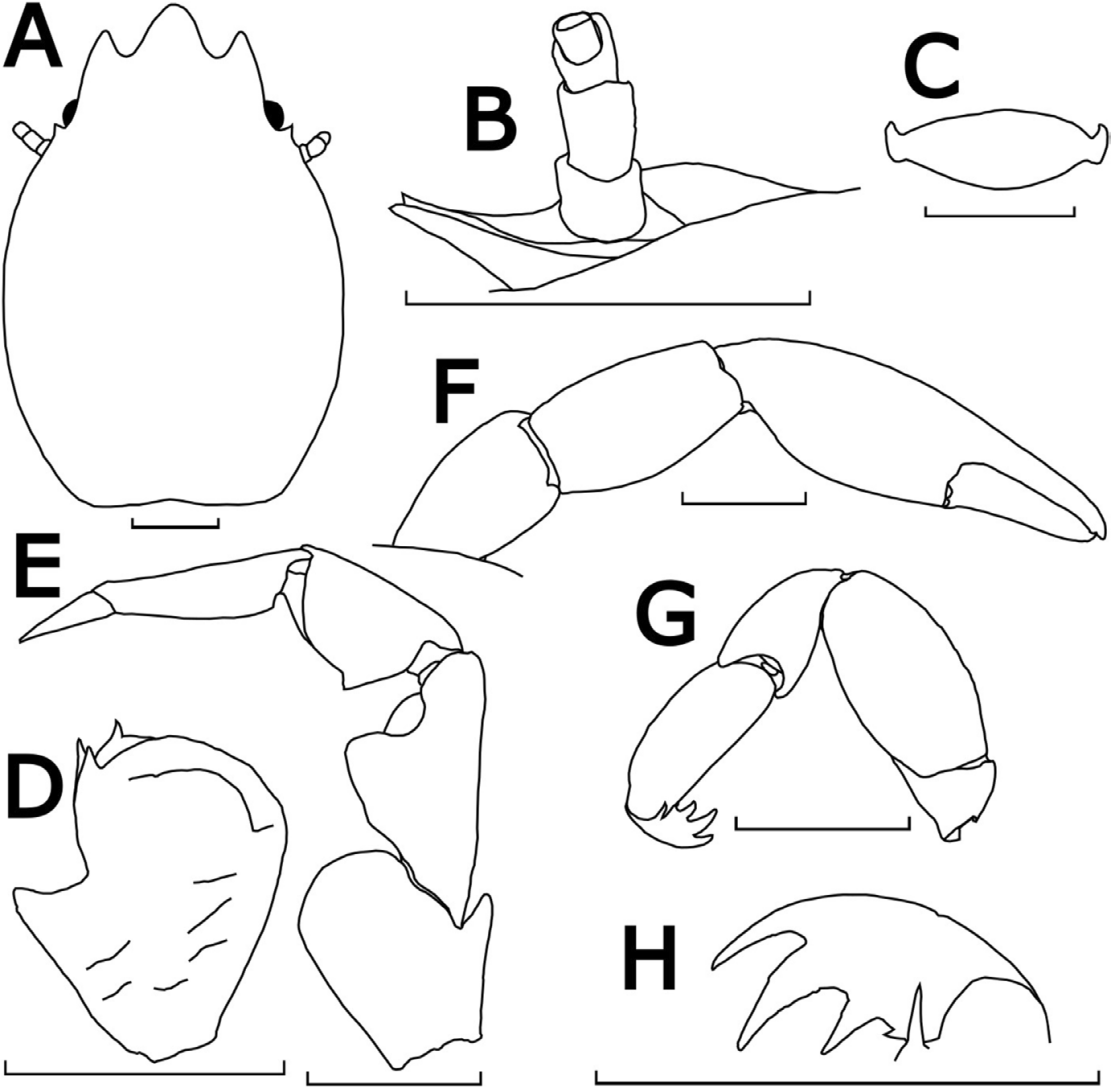
Morphological illustrations of *P. longiloba* n. sp., WAMC74721 (holotype). (A) Carapace, dorsal view; (B) upper margin of pterygostomian flap, posterior to left basal article of antennal peduncle, semi‐lateral view; (C) third thoracic sternite, ventral view; (D) left basal article of antennular peduncle, ventral view; (E) left third maxilliped, ventral view; (F) left cheliped, dorsal view; (G) left ambulatory leg (2nd pereopod), lateral view; (H) left dactylus of 2nd pereopod, dorsal view. Scale bar = 1 mm.

**FIGURE 13:**
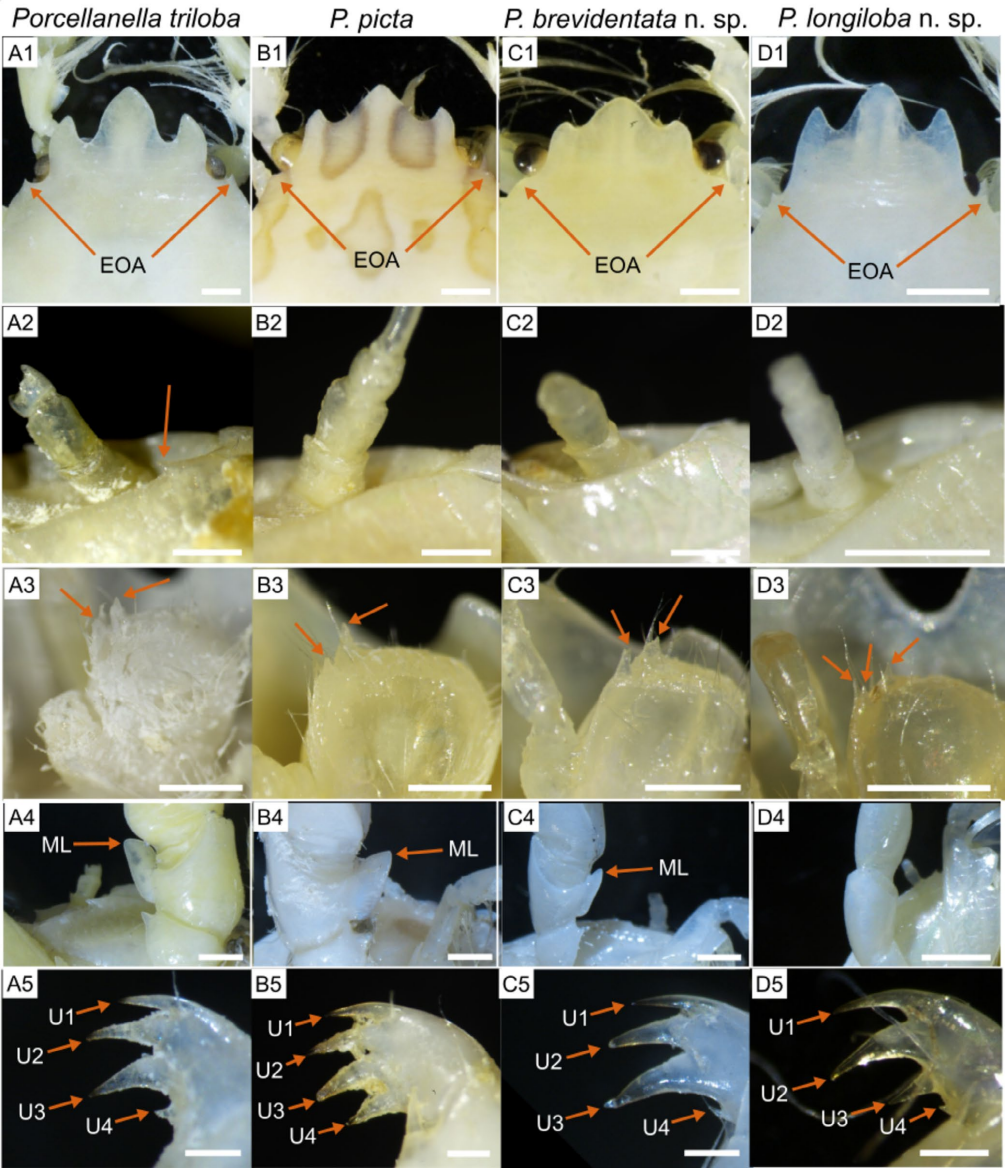
Comparisons between four *Porcellanella* species ((A) *P. triloba* ; (B) *P. picta* ; (C) *P. brevidentata* n. sp.; (D) *P. longiloba* n. sp.) of their distinct features ([1] Trilobate rostrum; [2] Upper margin of pterygostomian flap; [3] Basal article of antennular peduncle; [4] Cheliped merus; [5] Dactylus of ambulatory legs). Abbreviations: *EOA*, external orbital angles of carapace; *ML*, meral lobe of cheliped merus; *U1–4*, unguicles of dactylus from distal end. Scale bar: A1, A4, B1, B4, C1, C4, D1, D4 = 1.0 mm; A2–3, B2–3, C2–3, D2–3 = 0.5 mm; A5, B5, C5, D5 = 0.2 mm.

Type material: Holotype: **Western Australia** • WAMC74721 (Figure 8), collected on 16 May 2019 from Off Eighty Mile Beach (18°49′16.8″S, 120°19′26.3″ E) as the type locality. Paratype: **Western Australia** • WAMC40916, collected on 28 April 2006 from Ningaloo Marine Park (22°10′41.0″S, 113°47′33.0″ E).

Description: Carapace elongated, with average *CL*/*CW* ratio 1.55 (*n* = 2, Table S5). Rostrum extensively protruding with three teeth and *TRE*/*TRW* ratio 0.54 (*n* = 2, Table S5); median lobe triangular with semi‐rounded tip; lateral lobes extended sub‐parallel with median lobe. External orbital angles acute and slightly produced (Figures 12A and 13D1). Pterygostomian flaps unarmed on upper margin (Figures 12B and 13D2).

Third thoracic sternite attaching to third maxilliped thick, central process semi‐rounded with anterior margin relatively flattened, much larger than lateral lobes (Figure 12C).

Basal article of antennular peduncle longer than broad; anterior and lateral margins furnished with setae; ventral surface striated with setae; anterior plate much prolonged, with a single spinule at the distal‐mesial angle subequal in size with a pair of spinules at the medial‐mesial angle (Figures 12D and 13D3).

Antennal peduncle unarmed, basal article ventrally covered by pterygostomian flap (Figures 12B and 13D2).

Third maxilliped striated, bearing long plumose setae on mesial margins of carpus, propodus and dactylus (Figure 8B); ischium produced distally on extensor margin; merus as long as ischium, extensor margin straight, and flexor margin with subrectangular lobe; carpus triangular, divergent distally; propodus subequal with carpus in length (Figure 12E).

Chelipeds subequal; rather slender; surface smooth and glossy like carapace. Palm elongated, 3.33 times longer than broad, with line of dense plumose setae ventrally; dactylus to palm ratio 0.36. Carpi subcylindrical and relatively elongated, 1.74 times longer than broad. Meri without subtriangular lobe on dorsoflexor margin distally (Figures 12F and 13D4).

Ambulatory legs small, furnished with setae; merus longer than broad at a 1.90 ratio for 2nd pereopod (Figure 12G); propodus longer than broad at a 2.51 ratio for 2nd pereopod (Figure 12G), with a pair of spinules at the distal end of the posterior margin; dactylus armed with four sharp unguicles: second distinctly larger than third, fourth smallest and less than half of first (Figures 12H and 13D5).

Coloration in preservation: Overall body pale yellowish or white (Figure 8).

Etymology: The specific name is derived from the Latin ‘longus’ and ‘loba’, meaning ‘long rounded projection’, alluding to the rostral lobes, which are longer than those of other Australian *Porcellanella* species.

Distribution: Currently known from Off Eighty Mile Beach (type locality) and the Ningaloo Marine Park in Western Australia.

Habitat: The specimen WAMC74721 (holotype) was collected from a water depth of 75.2 m. The specimen WAMC40916 was collected from a water depth of 100 m.

Remarks: Among the three Australian lineages revealed from our phylogenetic analyses, this lineage with the longer extension of the trilobate rostrum was described as *P. longiloba* n. sp. (average *TRE*/*TRW* ratio = 0.54; ANOVA test *p* < 0.001; see Table S5 for measurement details and Tukey's comparison groupings).

*P. longiloba* n. sp. is distinguished from the other three species in this study ( *P. triloba* , *P. brevidentata* n. sp., and *P. picta* ) by the absence of a meral lobe on its cheliped's merus (Figure 13D4), whereas the other species possess this feature (Figure 13A4,B4,C4). Furthermore, the upper margin of its pterygostomian flap, posterior to the basal antennal peduncle, lacks a spinule (Figure 13D2), which differentiates it from *P. triloba* (Figure 13A2). The fourth distal unguicles on its ambulatory legs are very small, like half or less than half, the size of the first distal unguicle (Figure 13D5), which differentiates it from *P. picta* 's larger ones that are subequal to the first distal unguicle (Figure 13B5).

To differentiate between *P. longiloba* n. sp. and *P. haigae* , both of which lack a meral lobe on the cheliped merus (Figure S6), we can examine the median lobe shape of their trilobate rostrum, the external orbital angle and the basal article of their antennular peduncle: (1) The median rostrum of *P. longiloba* n. sp. has concave lateral margins and a rounded blunt tip (Figures 12A and 13D1). The illustration of *P. haigae* (Sankarankutty, 1963: figure 1a) depicted a median rostrum with convex lateral margins and an acute tip, whereas the ‘ *P. haigae* ’ illustrations from Werding and Hiller (2007: figure 18) and Tiwari et al. (2025): Figure 5A) depicted a median rostrum with relatively straight lateral margins and an acute tip (Figure S6); (2) The external orbital angles of *P* *. longiloba* n. sp. were relatively more extended and prominent (Figures 12A and 13D1) when compared to those of *P. haigae*, which were relatively less prominent (Sankarankutty 1963: figure 1a; Nakasone and Miyake, 1972: figure 3A; Tiwari et al. 2025: figures 2C and 5A); (3) For the antennular peduncle base, the anterior plate of *P. longiloba* n. sp. is produced into a distal‐mesial angle with a single spinule and a medial‐mesial angle with a pair of spinules that are subequal sizes, whereas the anterior plate of *P. haigae* (Sankarankutty, 1963: figure 1b) has two smaller spinules between the two mesial angles with relatively larger spinules. Additionally, *P. haigae* is notable for its transverse stripes on the chelipeds and vertical stripes on the carapace (Nakasone and Miyake 1972; online image: Chan and Lin 2013; online image: Ryanskiy n.d.), which can be referred to in future studies when a live specimen of *P. longiloba* n. sp. with colour markings is available for further comparison.

Although the *P. longiloba* n. sp. from Australia shares similar trilobate rostrum extension features (average TRE/TRW = 0.54, same grouping of Tukey's comparison for ANOVA *p* < 0.001) with the *P. haigae* illustrations from Gulf of Mannar (Sankarankutty 1963: figure 1a) and from Indonesia (Werding and Hiller 2007: figure 18a), it is different from the other ‘ *P. haigae* ’ (average TRE/TRW = 0.45, different grouping of Tukey's comparison for ANOVA *p* < 0.001) from Izu of Japan (Nakasone and Miyake 1972: figure 3a) and Papua New Guinea (online image: Chan and Lin 2013), as elaborated in the Table S5. By considering the wide geographical gap between the type localities of both species and the complication of *P. haigae* varieties, the *P. longiloba* n. sp. is argued to be a distinct species from the *P. haigae* with the support of morphological differences provided in this study, until future studies are able to provide more phylogenetic evidence for *P. haigae* .

### 3.7 Key to Species of Porcellanella White, (1851)

1a. Merus of cheliped without meral lobe on dorsoflexor margin distally …….…………….…………….…………….…………….…………….…………. 2.

1b. Merus of cheliped with meral lobe on dorsoflexor margin distally ………………………………………….…………….…………….…………….……… 3.

2a. Anterior plate of basal antennular peduncle produced into two mesial angles with 3 subequal‐sized spinules …………………………………………………………….…………….…………….…………*P. longiloba* n. sp.

2b. Anterior plate of basal antennular peduncle produced into two mesial angles with 2 relatively larger spinules and 2 relatively smaller spinules ……………….……… *P. haigae* Sankarankutty, (1963).

3a. Ambulatory leg dactylus with distal fourth unguicle subequal to distal first unguicle ……………………….… *P. picta* Stimpson, (1858).

3b. Ambulatory leg dactylus with distal fourth unguicle less than half the length of distal first unguicle ……………………………………… 4.

4a. Pterygostomian flap with spinule on upper margin posterior to antennal peduncle.……………………………… *P. triloba* White, (1851).

4b. Pterygostomian flap unarmed on upper margin ………………… ……………….…………….…………….…………….………… *P. brevidentata* n. sp.

## 4 Discussion

Overall, our study increases the number of recognised species in the genus *Porcellanella* from two (DecaNet 2024) to five. It highlights the importance of integrating molecular and morphological analyses to resolve long‐standing debates over the identification of cryptic species (Henderson 1893; Miyake 1943; Sankarankutty 1961; Johnson 1964; Ahyong et al. 2009; Osawa and McLaughlin 2010).

Our integrative molecular and morphological study indicates that *P. picta* is distinct from *P. triloba* in several key characteristics. Notably, *P. picta* is characterised by its large fourth distal unguicle of ambulatory legs (Figure 13B5), whereas in *P. triloba* this structure is much smaller (Figure 13A5); *P. triloba* possesses a tiny spinule at the upper margin of the pterygostomian flap posterior to the basal antennal peduncle (Figure 13A2), whereas such a feature is absence in *P. picta* (Figure 13B2). For the colour markings, *P. picta* exhibits oval to irregular colour patches on the chelipeds and the anterior dorsal carapace. Such markings are vivid in life, but faint markings can still be seen in specimens preserved over 20 years (i.e., ZRC2000.0907.1 and ZRC1998.0115.2). However, no colour markings are mentioned in the original description of *P. triloba* by White, (1851), or by Johnson (1964). All the *P. triloba* specimens examined in this study (QMC527357.1–3, QMC518947.1–2, WAMC74400) are all plain and without any trace of colour markings. From a molecular standpoint, the *COI* and *16S rRNA* sequences of *P. picta* show K2P distances of 17.57%–20.05% and 4.41%–7.28%, respectively, when compared to other species of *Porcellanella* (Table 3). These distances are significantly larger than the intraspecific variations, which range from 0.0% to 2.2% for *COI* and 0.0% to 1.4% for *16S rRNA* (Figure S4), therefore supporting the species distinctness of *P. picta* .

We identified samples of *Porcellanella* collected from Taiwan, Xiamen, Hong Kong, Thailand and Singapore as *P. picta* . Moreover, previous records of *P. picta* from Japan to Singapore (de Man 1888; Yokoya 1933; Miyake 1943; Johnson 1964) and records of ‘ *P. triloba* ’ from Taiwan to Celebes Sea (Henderson 1893; Haig 1992; Ahyong et al. 2009; Osawa and Chan 2010) all show characteristic traits that match our description of *P. picta* in this study, including the pigment patches on the chelipeds and anterior carapace, and the distinctly large fourth distal unguicle of ambulatory legs. Therefore, we suggest that *P. picta* is a widely distributed species along the Asian Pacific coasts, whereas those records of *P. triloba* from this region (Henderson 1893; Haig 1992; Ahyong et al. 2009; Osawa and Chan 2010) are incorrect. Consequently, we propose resurrecting *P. picta* to acknowledge its morphological and molecular distinctiveness, as well as its geographic distribution.

Previous studies also reported *Porcellanella* specimens collected from eastern Africa and the Indian Ocean as either *P. triloba* (Lens, 1905; Barnard, 1950; Macnae and Kalk 1958; Sankarankutty, 1961; Chowdhury and Mitra 2023) or *P. picta* (Sivasubramanian et al. 2014). The presence of pigment spots on the chelipeds and carapace and the robustness of the most proximal spinule of the dactylus of the ambulatory legs suggest these records are likely *P. picta* . However, given the long distance between these sampling locations and the nearest confirmed distribution range of *P. picta* in Thailand and Singapore, we call for caution in identifying these specimens before the availability of molecular sequences for comparison.

Our examination of ‘ *P. triloba* ’‐like specimens from Australia revealed three species. Morphologically, the *P. triloba* , *P. brevidentata* n. sp. and *P. longiloba* n. sp. can be distinguished by the shapes and extensions of the trilobate rostrum (Figure 13A1,C1,D1; Table S5), the presence of a spinule at the upper margin of the pterygostomian flap posterior to the basal antennal peduncle (Figure 13A2,C2,D2), and the presence of a cheliped meral lobe (Figure 13A4,C4,D4). Molecularly, these species exhibited interspecific K2P distances of 13.78%–18.63% for *COI* and 7.07%–8.20% for *16S rRNA* (Table 3), which also justifies their recognition as distinct species. Among the *P. triloba* specimens available for examination, the sampling sites included the Great Barrier Reef of eastern Australia (near the type locality of Cape Capricorn, Queensland) and Shark Bay of Western Australia, indicating their wide distribution in both the western and eastern Australian waters (Table 1). In contrast, the specimens of *P. brevidentata* n. sp. and *P. longiloba* n. sp. were both from western Australian waters only, indicating their more restricted distribution range. Nevertheless, given that our sample sizes for these three species are limited, further studies of ‘ *P. triloba* ’‐like specimens would likely expand their distribution ranges. Additionally, since we only examined preserved museum specimens for the species *P. triloba* , *P. brevidentata* n. sp. and *P. longiloba* n. sp., which lack vibrant colour and markings, and given that White, (1851) did not provide a description or illustration of live colours for the type specimen of *P. triloba* , future studies should investigate the colours and markings of fresh specimens for these respective species.

Our phylogenetic analyses serve as a starting point for discussing species divergence within *Porcellanella*. Notably, *P. triloba* , *P. bredidentata* n. sp. and *P. longiloba* n. sp. are all found around Australia, while *P. picta* is present along the Asia‐Pacific coasts. These trees suggest that the Australian waters are the centre of origin for this genus, whereas *P. picta* represents a more recent divergence. However, our phylogenetic analyses did not provide unequivocal support for sister relationships among the species. Depending on the genetic markers used, *P. picta* was found to be a sister to either *P. brevidentata* n. sp. or *P. longiloba* n. sp. Additional sequences from these species will help stabilise the tree topology and clarify the relationships of these species.

Future studies should also aim to obtain DNA sequences of *P. haigae* , which was first described from specimens collected in the Gulf of Mannar, Indian Ocean (Sankarankutty 1963). This species exhibits pigmentation on its chelipeds and carapace, but the pattern differs from that of *P. picta* , featuring transverse stripes on the chelipeds and vertical stripes on the carapace (Nakasone and Miyake 1972; online image: Chan and Lin 2013; online image: Ryanskiy, n.d.). *Porcellanella haigae* has been reported from locations ranging from Kenya and Madagascar in the western Indian Ocean to the Philippines and Papua New Guinea in the western Pacific (iNaturalist community, 2024). Additionally, a juvenile ‘ *P. haigae* ’ from Indonesia (Werding and Hiller 2007: figure 18) shows a different ratio of *TRE*/*TRW* compared to other *P. haigae* records (see Table S5). Given the wide distribution, we suggest that these records might represent a cryptic species complex. To test this hypothesis and place *P. haigae* within the phylogenetic framework of *Porcellanella*, future studies should obtain DNA data from geographically diverse populations.

## Author Contributions

**Hai Xin Loke:** data curation (lead), formal analysis (lead), writing – original draft (lead). **Bonnie Yuen Wai Heung:** data curation (equal), writing – review and editing (equal). **Yi‐Xuan Li:** data curation (equal), writing – review and editing (equal). **Yi‐Tao Lin:** data curation (equal), writing – review and editing (equal). **Andrew M. Hosie:** data curation (equal), validation (equal), writing – review and editing (equal). **Zhi Wang:** validation (equal), writing – review and editing (equal). **Marissa McNamara:** validation (equal), writing – review and editing (equal). **Jian‐Wen Qiu:** conceptualization (lead), funding acquisition (lead), resources (lead), supervision (lead), writing – review and editing (lead).

## Conflicts of Interest

The authors declare no conflicts of interest.

## Supporting information


**Figure S1:** Sampling stations of benthic biodiversity survey (supported by Lantau Conservation Fund, LCF/RE/2021/05) that recorded live specimen collection of *Porcellanella picta* during the period April 2022—March 2023.
**Figure S2:**. Four morphometrics of porcelain crab carapace. Abbreviations: *CL*, carapace length; *CW*, carapace width; *TRE*, trilobate rostrum extension; *TRW*, trilobate rostrum width.
**Figure S3:** Phylogenetic trees generated by Maximum Likelihood (ML) analyses for the (A) 569 bp *COI* and (B) 441 bp *16S rRNA* gene sequences of the *Porcellanella* of this study and outgroup. Values in parenthesis are the support values SH‐aLRT (%) / ultrafast bootstrap (UFBoot, %) of the nodes. Only UFBoot ≥ 70% are shown. GenBank accession numbers of the sequences used are listed in Table 2. The scale bar indicates the number of substitutions per site. Labelling of the Porcellanidae members followed Osawa and McLaughlin (2010).
**Figure S4:**. Pairwise comparisons of genetic distances (%) with Kimura 2‐parameter (K2P) between *Porcellanella* specimens for (A) 569 bp *COI* and (B) 441 bp *16S rRNA* sequences.
**Table S5:**. Carapace and trilobate rostrum measurements and ratios of the *Porcellanella* specimens. *Italicised numbers* are measurements taken in unit pixel due to image source without scale bar. Standard errors SE in parenthesis. Abbreviations: *CL*, carapace length; *CW*, carapace width; *TRE*, trilobate rostrum extension; *TRW*, trilobate rostrum width.
**Figure S6:**. Images of *Porcellanella longiloba* n. sp. (A–B), groups of *Porcellanella haigae* (C–G) and one suspected mislabelled porcelain crab (H) included for morphometric measurements. Sources of images: A (WAMC40916, this study); B (WAMC74721, this study); C (Sankarankutty, 1963); D (Werding and Hiller 2007); E (online image: Chan and Lin 2013); F (Nakasone and Miyake 1972); G (Ryanskiy, n.d.); H (Miyake 1943).
**Figure S7:**. Three *Porcellanella picta* individuals showcase similar pale yellowish or white colour on overall body, but display irregular shaped spots or ocelli markings on dorsal side of arms and carapace anterior portion. (A) individual one; (B) individual two; (C) dorsal and (D) ventral view of individual three. Scale bar: A–D = 10.0 mm. Location: Northwest of Lantau Island, Hong Kong. Date: 6 March 2024.

## Data Availability

This paper is registered in ZooBank (urn:lsid:zoobank.org:pub:E9B897B6‐7BA2‐4221‐9A7B‐D9D06B13422D). Sequences of the porcelain crab gene fragments were deposited in GenBank under the accession numbers PQ856281 to PQ856308 (*COI*) and PQ865372 to PQ865398 (*16S rRNA*).
