# Supplementary material for: Integrative Phylogenetic and Morphological Analyses Reveal Two New Species of Porcellanid Crabs and Resurrect Porcellanella picta Stimpson, 1858 (Decapoda: Porcellanidae)
Source: Ecol Evol. 2025 Sep 29;15(10):e72131. doi: 10.1002/ece3.72131 (PMC12480436; doi:10.1002/ece3.72131)
Supplement: Supplementary file 1 — Figure S1: Sampling stations of benthic biodiversity survey (supported by Lantau Conservation Fund, LCF/RE/2021/05) that recorded live specimen collection of Porcellanella picta during the period April 2022—March 2023. Figure S2:. Four morphometrics of porcelain crab carapace. Abbreviations: CL, carapace length; CW, carapace width; TRE, trilobate rostrum extension; TRW, trilobate rostrum width. Figure S3: Phylogenetic trees generated by Maximum Likelihood (ML) analyses for the (A) 569 bp COI and (B) 441 bp 16S rRNA gene sequences of the Porcellanella of this study and outgroup. Values in parenthesis are the support values SH‐aLRT (%) / ultrafast bootstrap (UFBoot, %) of the nodes. Only UFBoot ≥ 70% are shown. GenBank accession numbers of the sequences used are listed in Table 2. The scale bar indicates the number of substitutions per site. Labelling of the Porcellanidae members followed Osawa and McLaughlin (2010). Figure S4:. Pairwise comparisons of genetic distances (%) with Kimura 2‐parameter (K2P) between Porcellanella specimens for (A) 569 bp COI and (B) 441 bp 16S rRNA sequences. Table S5:. Carapace and trilobate rostrum measurements and ratios of the Porcellanella specimens. Italicised numbers are measurements taken in unit pixel due to image source without scale bar. Standard errors SE in parenthesis. Abbreviations: CL, carapace length; CW, carapace width; TRE, trilobate rostrum extension; TRW, trilobate rostrum width. Figure S6:. Images of Porcellanella longiloba n. sp. (A–B), groups of Porcellanella haigae (C–G) and one suspected mislabelled porcelain crab (H) included for morphometric measurements. Sources of images: A (WAMC40916, this study); B (WAMC74721, this study); C (Sankarankutty, 1963); D (Werding and Hiller 2007); E (online image: Chan and Lin 2013); F (Nakasone and Miyake 1972); G (Ryanskiy, n.d.); H (Miyake 1943). Figure S7:. Three Porcellanella picta individuals showcase similar pale yellowish or white colour on overall body, but display irreg [file ECE3-15-e72131-s001.docx]

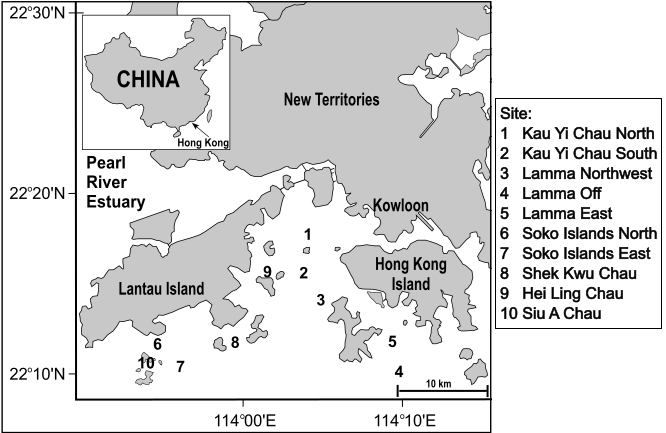


**Supplementary Figure S1**. Sampling stations of benthic biodiversity survey (supported by Lantau Conservation Fund, LCF/RE/2021/05) that recorded live specimen collection of *Porcellanella picta* during the period April 2022 – March 2023.


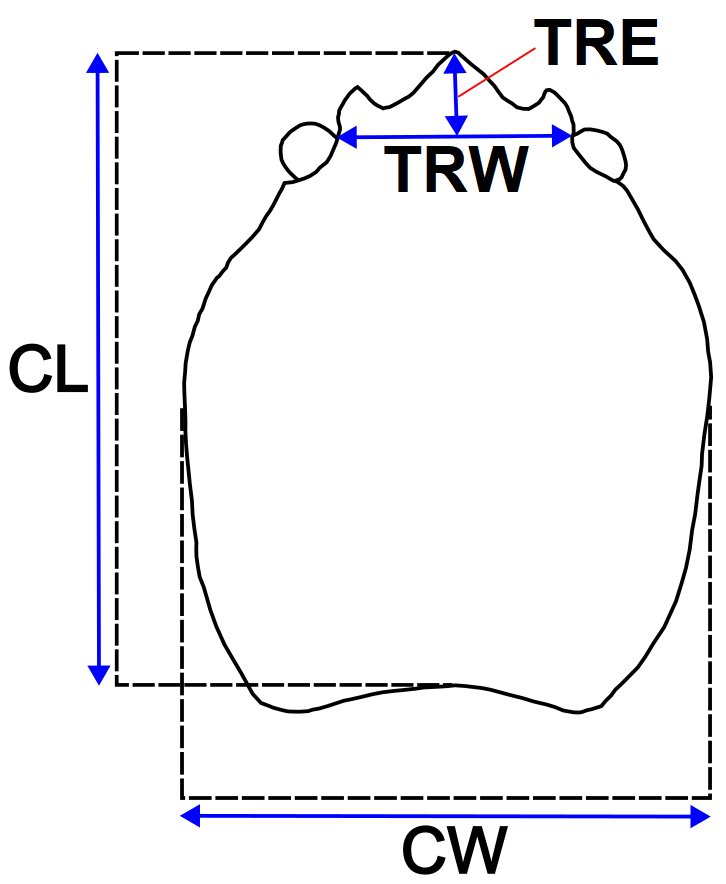


**Supplementary Figure S2**. Four morphometrics of porcelain crab carapace. Abbreviations: *CL*, carapace length; *CW*, carapace width; *TRE*, trilobate rostrum extension; *TRW*, trilobate rostrum width.


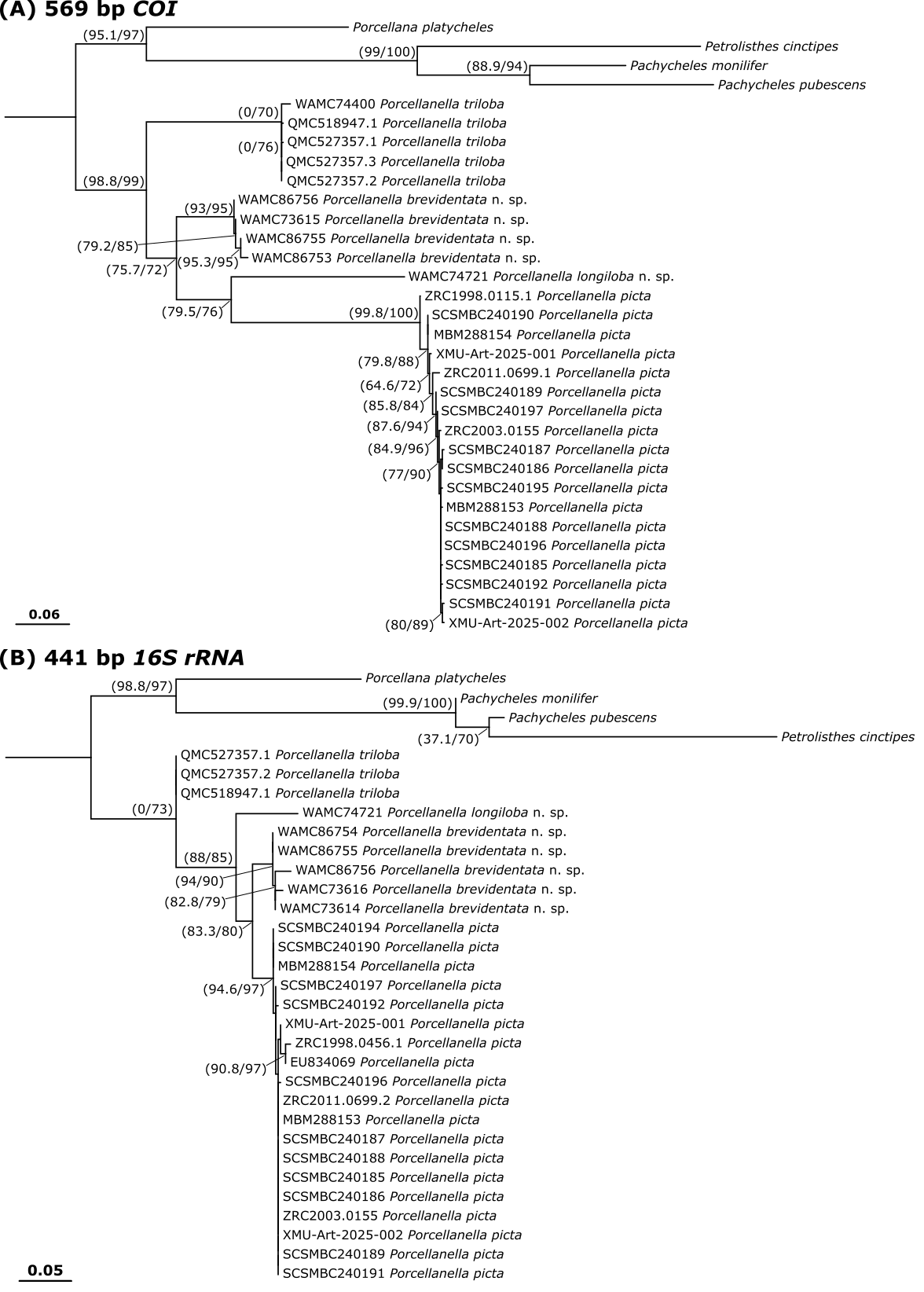


**Supplementary Figure S3.** Phylogenetic trees generated by Maximum Likelihood (ML) analyses for the (A) 569 bp *COI* and (B) 441 bp *16S rRNA* gene sequences of the *Porcellanella* of this study and outgroup. Values in parenthesis are the support values SH-aLRT (%) / ultrafast bootstrap (UFBoot, %) of the nodes. Only UFBoot ≥ 70% are shown. GenBank accession numbers of the sequences used are listed in Table 2. The scale bar indicates the number of substitutions per site. Labelling of the Porcellanidae members followed Osawa and McLaughlin (2010).

**Supplementary Figure S4.** Pairwise comparisons of genetic distances (%) with Kimura 2-parameter (K2P) between *Porcellanella* specimens for (A) 569 bp *COI* and (B) 441 bp *16S rRNA* sequences.

**(cont.) Supplementary Figure S4.** Pairwise comparisons of genetic distances (%) with Kimura 2-parameter (K2P) between *Porcellanella* specimens for (A) 569 bp *COI* and (B) 441 bp *16S rRNA* sequences

**Supplementary Table S5**. Carapace and trilobate rostrum measurements and ratios of the *Porcellanella* specimens. *Italicised numbers* are measurements taken in unit pixel due to image source without scale bar. Standard errors SE in parenthesis. Abbreviations: *CL*, carapace length; *CW*, carapace width; *TRE*, trilobate rostrum extension; *TRW*, trilobate rostrum width.

| Specimen image source | *CL*, mm | *CW*, mm | *CL/CW* ratio | Average (SE) *CL/CW* ratio | *TRE*, mm | *TRW*, mm | *TRE/TRW* ratio | Average (SE) *TRE/TRW* ratio |
| --- | --- | --- | --- | --- | --- | --- | --- | --- |
| *Porcellanella triloba* | | | | | | | | |
| QMC518947.1 | 8.12 | 6.31 | 1.29 | 1.34 (0.01) ^B, C^ | 1.18 | 2.99 | 0.40 | 0.434 (0.012) ^Y^ |
| QMC518947.2 | 5.00 | 3.68 | 1.36 |  | 0.91 | 1.96 | 0.46 |  |
| QMC527357.1 | 8.24 | 6.03 | 1.37 |  | 1.31 | 3.16 | 0.41 |  |
| QMC527357.2 | 7.31 | 5.51 | 1.33 |  | 1.24 | 2.87 | 0.43 |  |
| QMC527357.3 | 8.70 | 6.35 | 1.37 |  | 1.51 | 3.19 | 0.47 |  |
| WAMC74400 | 11.03 | 8.35 | 1.32 |  | 1.72 | 4.04 | 0.43 |  |
| *Porcellanella picta* | | | | | | | | |
| SCSMBC240192 | 8.61 | 7.13 | 1.21 | 1.26 (0.02) ^D^ | 1.12 | 3.24 | 0.35 | 0.332 (0.005) ^Z^ |
| SCSMBC240193 | 9.68 | 7.83 | 1.24 |  | 1.13 | 3.60 | 0.31 |  |
| SCSMBC240194 | 9.62 | 7.96 | 1.21 |  | 1.26 | 3.47 | 0.36 |  |
| SCSMBC240195 | 9.22 | 7.49 | 1.23 |  | 1.12 | 3.31 | 0.34 |  |
| SCSMBC240196 | 8.53 | 6.71 | 1.27 |  | 0.93 | 3.01 | 0.31 |  |
| SCSMBC240198 | 6.45 | 5.35 | 1.20 |  | 0.81 | 2.39 | 0.34 |  |
| SCSMBC240199 | 5.42 | 4.57 | 1.19 |  | 0.75 | 2.10 | 0.35 |  |
| SCSMBC240200 | 7.53 | 6.03 | 1.25 |  | 0.94 | 2.65 | 0.36 |  |
| ZRC1998.0456.1 | 10.21 | 7.94 | 1.29 |  | 1.11 | 3.75 | 0.30 |  |
| ZRC2000.0907.1 | 9.90 | 7.36 | 1.34 |  | 1.14 | 3.51 | 0.33 |  |
| ZRC2003.0155 | 9.08 | 7.39 | 1.23 |  | 1.12 | 3.26 | 0.34 |  |
| ZRC1998.0113 | 7.32 | 5.79 | 1.26 |  | 0.83 | 2.73 | 0.31 |  |
| ZRC1998.0115.1 | 5.92 | 4.37 | 1.36 |  | 0.73 | 2.16 | 0.34 |  |
| ZRC1998.0115.2 | 5.55 | 4.02 | 1.38 |  | 0.73 | 2.14 | 0.34 |  |
| ZRC2011.0699.1 | 7.39 | 6.18 | 1.20 |  | 0.88 | 2.86 | 0.31 |  |
| *Porcellanella brevidentata* n. sp. | | | | | | | | |
| WAMC44990.1 | n/a | 6.27 | n/a | 1.27 (0.01) ^C, D^ | n/a | 3.20 | n/a | 0.340 (0.008) ^Z^ |
| WAMC44990.2 | 7.50 | 5.88 | 1.28 |  | 1.14 | 2.81 | 0.41 |  |
| WAMC73614 | 6.97 | 5.59 | 1.25 |  | 0.82 | 2.47 | 0.33 |  |
| WAMC73615 | 7.16 | 5.61 | 1.28 |  | 0.83 | 2.68 | 0.31 |  |
| WAMC86753 | 6.39 | 5.12 | 1.25 |  | 0.82 | 2.42 | 0.34 |  |
| WAMC73616 | 7.68 | 5.78 | 1.33 |  | 0.91 | 2.78 | 0.33 |  |
| WAMC79506.1 | 6.23 | 4.92 | 1.27 |  | 0.87 | 2.49 | 0.35 |  |
| WAMC79506.2 | 5.76 | 4.56 | 1.26 |  | 0.79 | 2.29 | 0.34 |  |
| WAMC86754 | 6.76 | 5.34 | 1.27 |  | 0.90 | 2.69 | 0.33 |  |
| WAMC86755 | 7.97 | 6.31 | 1.26 |  | 1.00 | 3.00 | 0.33 |  |
| WAMC86756 | 8.26 | 6.52 | 1.27 |  | 1.03 | 3.20 | 0.32 |  |
| *Porcellanella longiloba* n. sp. | | | | | | | | |
| WAMC40916 | 6.59 | 4.16 | 1.59 | 1.55 (0.04) ^A^ | 1.31 | 2.34 | 0.56 | 0.539 (0.019) ^W^ |
| WAMC74721 | 5.72 | 3.80 | 1.51 |  | 1.09 | 2.09 | 0.52 |  |
| *Porcellanella haigae* (holotype) |  |  |  |  |  |  |  |  |
| Sankarankutty, 1963 | n/a | n/a | n/a |  | 120.01 | 221.90 | 0.54 | 0.541 ^W^ |
| "*Porcellanella haigae*" group 1 (likely) | | | | | | | | |
| Werding & Hiller, 2007 | 3.47 | 2.21 | 1.58 | 1.58 ^A^ | 0.72 | 1.34 | 0.54 | 0.535 ^W, X^ |
| "*Porcellanella haigae*" group 2 (unlikely) | | | | | | | | |
| Chan & Lin, 2013 | *238.29* | *165.38* | 1.44 | 1.43 (0.00) ^A, B^ | *45.56* | *97.42* | 0.47 | 0.446 (0.012) ^X, Y^ |
| Nakasone & Miyake, 1972 | *539.43* | *376.46* | 1.43 |  | *97.08* | *218.02* | 0.45 |  |
| Ryanskiy, n.d. | *404.85* | *284.03* | 1.43 |  | *66.72* | *156.92* | 0.43 |  |
| *Porcellanella* sp. n.d. (not *P. triloba*) | | |  |  |  |  |  |  |
| Miyake, 1943 | *572.72* | *361.53* | 1.58 | 1.58 ^A^ | *117.11* | *204.00* | 0.57 | 0.57 ^W^ |

Note: ANOVA tests showed significant differences in both *CL*/*CW* (*F* = 28.57, *P* < 0.001) and *TRE*/*TRW* (*F* = 53.88, *P* < 0.001). Mean ratios that do not share a superscripted letter are significantly different (Tukey’s comparisons groupings: A–D for *CL*/*CW*; W–Z for *TRE*/*TRW*).


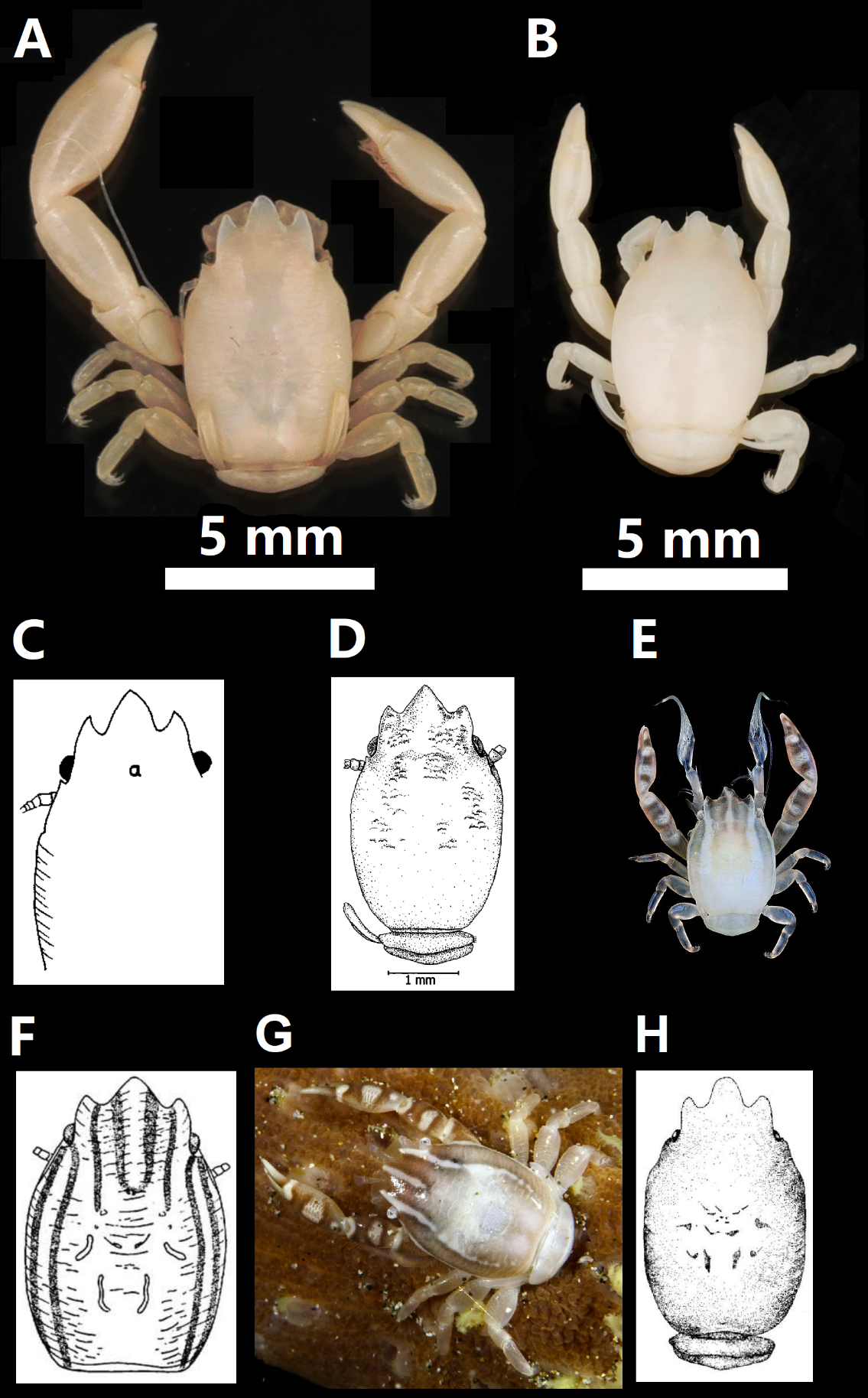


**Supplementary Figure S6**. Images of *Porcellanella longiloba* n. sp. (A–B), groups of *Porcellanella haigae* (C–G), and one suspected mislabelled porcelain crab (H) included for morphometric measurements. Sources of images: A (WAMC40916, this study); B (WAMC74721, this study); C (Sankarankutty, 1963); D (Werding & Hiller, 2007); E (online image: Chan & Lin, 2013); F (Nakasone & Miyake, 1972); G (online image: Ryanskiy, n.d.); H (Miyake, 1943).


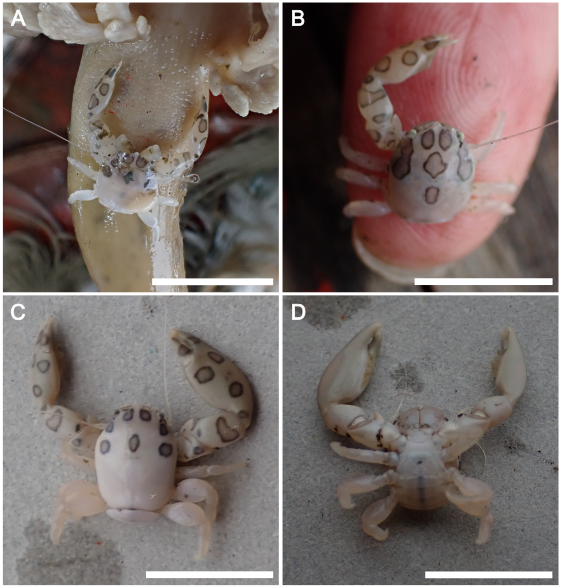


**Supplementary Figure S7**. Three *Porcellanella picta* individuals showcase similar pale yellowish or white colour on overall body, but display irregular shaped spots or ocelli markings on dorsal side of arms and carapace anterior portion. (A) individual one; (B) individual two; (C) dorsal and (D) ventral view of individual three. Scale bar: A–D = 10.0 mm. Location: Northwest of Lantau Island, Hong Kong. Date: 6 March 2024.
